# Cross-Country Comparisons of Covid-19: Policy, Politics and the Price of Life

**DOI:** 10.1007/s10640-020-00466-5

**Published:** 2020-08-04

**Authors:** Ben Balmford, James D. Annan, Julia C. Hargreaves, Marina Altoè, Ian J. Bateman

**Affiliations:** 1 Department of Economics, Land, Environment Economics and Policy Institute, University of Exeter Business School, Exeter, EX4 4PU UK; 2 Blue Skies Research, Ltd, Settle, UK; 3 Innovation, Impact and Business, University of Exeter, Exeter, UK

**Keywords:** Coronavirus, Covid-19, Modeling, Valuation, Price of life

## Abstract

Coronavirus has claimed the lives of over half a million people world-wide and this death toll continues to rise rapidly each day. In the absence of a vaccine, non-clinical preventative measures have been implemented as the principal means of limiting deaths. However, these measures have caused unprecedented disruption to daily lives and economic activity. Given this developing crisis, the potential for a second wave of infections and the near certainty of future pandemics, lessons need to be rapidly gleaned from the available data. We address the challenges of cross-country comparisons by allowing for differences in reporting and variation in underlying socio-economic conditions between countries. Our analyses show that, to date, differences in policy interventions have out-weighed socio-economic variation in explaining the range of death rates observed in the data. Our epidemiological models show that across 8 countries a further week long delay in imposing lockdown would likely have cost more than half a million lives. Furthermore, those countries which acted more promptly saved substantially more lives than those that delayed. Linking decisions over the timing of lockdown and consequent deaths to economic data, we reveal the costs that national governments were implicitly prepared to pay to protect their citizens as reflected in the economic activity foregone to save lives. These ‘price of life’ estimates vary enormously between countries, ranging from as low as around $100,000 (e.g. the UK, US and Italy) to in excess of $1million (e.g. Denmark, Germany, New Zealand and Korea). The lowest estimates are further reduced once we correct for under-reporting of Covid-19 deaths.

**Electronic supplementary material:**

The online version of this article (10.1007/s10640-020-00466-5) contains supplementary material, which is available to authorized users.

## Introduction

SARS-CoV-2, the virus which causes the Covid-19 disease, is a zoonotic pathogen which emerged in Wuhan in late 2019 (Huang et al. 2020). At the time of writing, in early July 2020, it had already claimed the lives of over half a million people globally (Beltekian et al. 2020). In the USA Covid-19 deaths now exceed the number of US military deaths arising from all conflict since the Second World War (Statista 2020) while in the UK the four weeks to 24th April saw more Londoners lose their lives to Covid-19 than during the deadliest four week period of the Blitz (Morris and Barnes 2020).

This death toll is only the extremely saddening tip of the much larger iceberg of disruption that Covid-19 has caused and continues to cause. Confirmed cases across the world now exceed eleven million (Beltekian et al. 2020) and the true infection rate is likely far higher. Each case imposes a real cost on every infected individual. While symptoms may sound innocuous, including a dry cough, fever, and tiredness (WHO 2020a; Verity et al. 2020), longer term this morbidity is likely to impose significant costs on sufferers’ health, including potentially permanent lung damage or fibrosis associated with impacts upon the heart, kidneys and brain (Citroner 2020), all of which are likely to have negative consequences for future well-being and productivity.

Moreover, alongside the vast disruption that the virus itself has caused directly, preventative measures have caused further disarray in the economy. At present, there are no known specific treatments or available vaccines to either cure or prevent Covid-19 infections (WHO 2020b). Therefore governments world-wide have relied upon preventative measures which aim to reduce the number of people exposed to the virus, and lower the effective reproductive number (the average number of new cases per infection, known as R), ideally suppressing it below a value of 1 at which point the number of active cases decreases over time (Ferguson et al. 2020). While some of these measures impose relatively little personal or economic cost (such as simple hand hygiene and the use of face masks), the failure of such measures to stem the rapid world-wide spread of the virus has necessitated international “stay at home” lockdown requirements, entailing significant impacts across the global economy. The International Monetary Fund (IMF) predicts a contraction in global GDP of three percent in 2020—a decline of 6.4% relative to its October 2019 forecast—and a decrease which it describes as being “much worse than during the 2008–2009 financial crisis” (IMF 2020a). Short term effects are even more extreme. For example, in the UK, GDP fell by 20.4% in April 2020 (ONS 2020a), while those claiming unemployment benefits rose nearly 70% to over 2 million (ONS 2020b), although even this is dwarfed by the 200% increase in US unemployment over the same period (Aratani 2020).^1^ Globally sovereign debt is also soaring: predicted to grow nearly 20% to $53 trillion in 2020 (Standard and Poor 2020) as administrations around the world race to protect cash-strapped companies from going out of business in order to prevent further unemployment.

At the human level, lives and livelihoods have been turned upside-down. Hence the true economic costs are more diverse and quite possibly more severe than that captured by financial metrics alone. They include negative ramifications for people’s mental health (Pancani et al. 2020; Chaix et al. 2020; Branley-Bell and Talbot 2020); increased prevalence of domestic violence (McLay 2020); and likely reduce the educational achievement of today’s children (Pinto and Jones 2020; Van Lancker and Parolin 2020). As with previous financial crises (Hoynes et al. 2012) and pandemics (Nikolopoulos et al. 2011), the virus and the economic fall-out are disproportionately affecting people from disadvantaged groups and lower-income households. Black, Asian and Minority Ethnic people are more likely to be infected and die (Bhala et al. 2020; Garg 2020; Khunti et al. 2020; Yancy 2020; Public Health England 2020); and lower-income households are less likely to be able to work from home, so face greater negative income shocks (Hanspal et al. 2020; Hensvik et al. 2020), just as poorer countries are likely to suffer more than richer nations (Hevia and Neumeyer 2020).

As is well known, different countries have had very different death tolls. The USA currently has the highest death toll in the world, already exceeding 130,000 deaths (as of 4th July 2020).^2^ In contrast, Vietnam—which recorded its first case just 4 days after the USA—is yet to experience a single death. Understanding what drives these differences is clearly crucial, potentially enabling improved responses to the continuing Covid-19 outbreak and future pandemics. This paper begins to answer the critical question of why different countries have suffered different death rates, and what we can learn for future policy.

The remainder of the paper is set out as follows. In Sect. 2 we first compare the numbers of deaths attributed to Covid-19 across all OECD countries. The paper briefly focusses upon the UK as an example of a broader pattern; that public reporting of numbers related to the pandemic can be somewhat misleading. Next, we control for any within-country under-reporting by analysing the overall increase in all deaths above what would be seasonally expected. Assessing these ‘excess deaths’ data suggests that in most nations for which information is available official reporting of Covid-19 tends to explain most of this unexpected mortality. However, analysis also reveals some clear exceptions, such as in the Netherlands, Spain and the UK where more than 40% of all Covid-19 deaths seem likely to have not been counted as such. Addressing such reporting problems is an essential element of providing the informational base required for an evidence-based policy response to this and any future pandemics.

In Sect. 3 we assess the impact of government decisions regarding lockdown, their effectiveness and the policy trade-off between economic activity and health risk that they reveal. Accepting that they are a conservative estimate of the total impact of the pandemic, officially attributed Covid-19 deaths are used to investigate the price of life implied by lockdown policies. First we use a simple regression analysis to show that differences in mortality rates between countries are not driven by factors which are beyond the short term control of policy makers—such as differences in income and equality which, at least within the time available to fight coronavirus are effectively fixed. This in turn allows us to examine the degree of control which policymakers do have at their disposal, such as the rapidity of lockdown imposition and the duration of such controls. We use country-specific Susceptible-Exposed-Infected-Recovered (SEIR) models, similar to the approach of Ferguson et al. (2020), to ask how changes in the timing of lockdown measures affect the current death toll. Our analyses provide good evidence that these policy tools actually determine the majority of variation in Covid-19 impacts between countries. Finally, we link these estimates to financial data to reveal a huge variation in the implied price of life across countries. Section 4 concludes.

## The Current Global Situation: Reconciling Different Measures

### Cross-Country Differences

Table 1 presents the number of tests, cases and deaths that are officially recorded as (at least in part) caused by Covid-19 across all OECD countries as of 9th June 2020 (data from Our World in Data; Beltekian et al. 2020). As mentioned, and considered in greater detail subsequently, these official estimates are likely to under-estimate deaths from Covid-19. However, the degree of under-reporting is far from constant across countries. For example, while almost all countries only counted deaths which had been confirmed to be linked to Covid-19, Belgium adopts a much broader approach also including deaths where Covid-19 is merely suspected as a contributory factor (Chini 2020). This results in much higher death rates than in other countries. Arguably adopting the Belgian approach internationally might provide a more accurate picture of Covid-19 mortality.

**Table 1:** Covid-19 data for OECD countries

Country	Total Covid-19 tests (thousands)	Tests per million	Total recorded Covid-19 cases	Cases per million	Total Covid-19 attributed deaths	Deaths per million
Australia	1579	61,930	7270	285	102	4
Austria	490	54,360	16,890	1875	672	75
Belgium	741	63,980	59,350	5121	9609	829
Canada	1868	49,500	96,230	2550	7835	207
Chile	688	35,960	138,850	7263	2264	118
Colombia	400	7850	40,720	800	1308	26
Czech Rep.	472	44,030	9700	906	328	31
Denmark	572	98,720	11,960	2065	593	102
Estonia	90	67,900	1940	1462	69	52
Finland	201	36,290	7000	1264	323	58
France	NA	NA	154,190	2362	29,209	447
Germany	4349	51,910	184,540	2203	8711	104
Greece	194	18,610	3050	293	182	17
Hungary	207	21,410	4010	416	548	57
Iceland	63	183,820	1810	5295	10	29
Ireland	348	70,560	25,210	5105	1683	341
Israel	565	65,290	18,090	2090	298	34
Italy	4187	69,250	235,280	3891	33,964	562
Japan	314	2490	17,210	136	916	7
Korea	1013	19,750	11,850	231	274	5
Latvia	118	62,380	1090	577	26	14
Lithuania	326	119,860	1720	632	71	26
Luxembourg	85	135,680	4040	6454	110	176
Mexico	284	2200	120,100	932	14,053	109
Netherlands	392	22,850	47,740	2786	6016	351
New Zealand	294	60,980	1150	239	22	5
Norway	257	47,460	8550	1577	239	44
Poland	967	25,560	27,160	718	1166	31
Portugal	874	85,710	34,890	3421	1485	146
Slovakia	188	34,480	1530	280	28	5
Slovenia	83	39,860	1490	714	108	52
Spain	2536	54,250	241,720	5170	27,136	580
Sweden	276	27,280	45,130	4469	4694	465
Switzerland	423	48,930	30,890	3569	1660	192
Turkey	2303	27,310	171,120	2029	4711	56
UK	3250	47,880	287,400	4234	40,597	598
USA	20,385	61,590	1,961,190	5925	111,007	335

Data from Our World in Data, correct as of 9th June 2020. Tests are to the nearest thousand, measurement unit varies by country. Tests per million to the nearest tens of tests. Total cases to the nearest ten cases, cases per million population to the nearest whole number. Deaths, both total and per million, to the nearest whole number

It is worth drawing attention to the very substantial variation in tests, recorded Covid-19 case numbers and official death tolls across countries. Adjusting for population, Iceland has undertaken far more testing per capita than any other OECD country, at over 183 K/million compared to just 2 K/million in Mexico.

Much media attention has been expended upon reporting cumulative Covid-19 numbers in each country. In terms of cases the roughly 2 million cases reported in the USA is indeed a prominent result. However, unsurprisingly it is the total numbers of deaths by country which has attracted more attention and again the US total of well over 100,000 deaths is eye-catching. However, this media and policy-maker focus upon totals disguises the true comparison of these figures in failing to make even the most basic of adjustments for variation in population size between countries. Once this is done then the death rate per million shown in the final column of Table 1 reveals a substantially different story. Here we need to rule Belgium out of comparison because its addition of suspected Covid-19 deaths to the confirmed deaths reported by other countries, upwardly inflates its death rate. Given this, the death rate reported in the UK is the highest amongst all of the OECD, exceeding even those of Spain and Italy which experienced their first major outbreaks much earlier on in the pandemic.

**Fig. 1:**
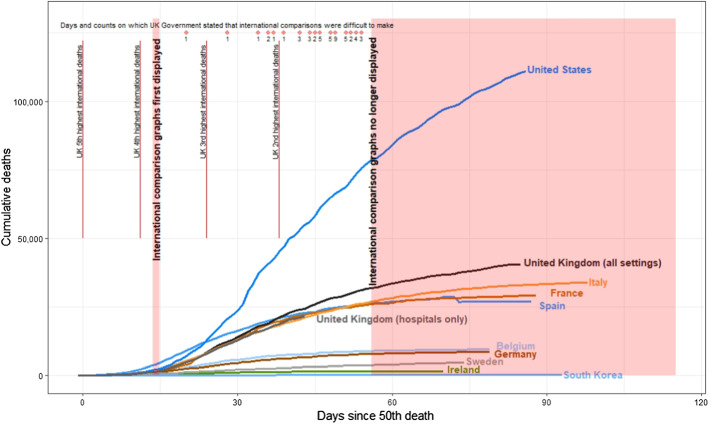
Cumulative deaths (vertical axis) plotted for various countries (as selected for comparison in UK Government briefings) over approximately the first 100 days since each country recorded its fiftieth death (horizontal axis). Note that Spain’s apparent decrease in cumulative deaths around day 70 is an artefact of their reporting problems

It is worth highlighting how reporting elsewhere can be somewhat misleading. We do so by focussing on the UK as this is the country we are most familiar with, but the story is highly likely to be similar elsewhere. Figure 1 graphs the development of total recorded deaths (vertical axis) for a selection of 10 countries over roughly the first 100 days since each country recorded its 50th death (horizontal axis). This graph and its selection of countries is dictated by that which the UK government chose to highlight for comparison at its daily coronavirus press briefings.^3^

Setting aside for the moment the US trend, clear separation can be observed between those countries such as Germany and Korea, which rapidly entered into lockdown and quickly controlled the growth of the virus, and those countries such as the UK and Spain, where lockdown was delayed resulting in a higher plateau. This is the first indication of the positive effects of early lockdown action, which we consider further subsequently.

The UK government’s decision to only display the total number of deaths in each of the countries shown took no account of even basic differences between countries such as population size; and as Table 1 has already shown, this makes fair comparison of death rates difficult. It might seem unusual to fail to make such basic adjustments, however the choice of such a display by the government is one which shows the UK cumulative total initially below that of European neighbours such as Italy and Spain and consistently dwarfed by that for the US, rising to more than twice the UK level. The fact that the US population is more than five times that of the UK, and that therefore per capita rates were much higher in the UK, is not obvious in this display.

During the early days of the coronavirus outbreak, this omission of per capita data and focus upon cumulative totals allowed the UK government to make cross country comparisons which indicated that the country appeared to be faring better than many international counterparts (such sentiments are clear in transcripts of the verbal explanation which accompanied the graph, presented in Online Appendix 1). For example, on the 1st April, the graph was described by the UK government as showing “it has not been as severe here as in France, and we are just tucked in under the USA and obviously Italy on a different trajectory”. However, as the pandemic developed so the performance of the UK relative to these other countries worsened. This situation was exacerbated by an outcry against the UK government’s use of statistics based only upon deaths within hospitals rather than also including those in the community, ignoring obvious discrepancies such as a clear rise in deaths within care homes into which elderly hospital patients had been moved without testing for coronavirus (Discombe 2020; Grey and MacAskill 2020). Shifting to reporting deaths from all settings revealed that the UK was faring far worse than nearly all other countries and indeed in per capita terms was experiencing one of the highest death rates globally (Beltekian et al. 2020).

The impact upon the official narrative presented at UK press briefings was swift and noticeable. While initially much emphasis had been placed upon the UK’s apparently favourable performance compared to other nations, now Government officials started to mention the difficulty of making cross country comparisons, as highlighted by the pink dots at the top of Fig. 1 (and data presented in Online Appendix 2).^4^ These caveats increased in both regularity and stridency until, on 10th May 2020, cross country comparisons were removed from Government press conferences. We have no reason to suspect that the UK government was unique in attempting to provide a positive representation of trends. However, a failure to provide clear and objective information is a well acknowledged cause of mistrust in authority (Kavanagh and Rich 2018) and is corrosive to public life at any time, but particularly in a pandemic where trust in institutions is vital.

### Challenges to Making Cross-Country Comparisons

In undertaking cross-country comparisons of the impacts of Covid-19 a first issue to be tackled is the difference in national approaches to reporting. This can be seen even in the reporting of testing statistics, differences which some authorities have argued may be politically motivated (Norgrove 2020). Likewise, some countries (e.g. Belgium) are far more likely than others to ascribe a death as caused by Covid-19 (Chini 2020).

Given these concerns, we complement our comparisons of official Covid-19 statistics with analysis of patterns in excess mortality data. Here we define excess mortality for a country as the deviation in mortality rate during the period January to April 2020 compared to a baseline of expected deaths from previous years. Excess mortality data is therefore not biased by differential rates of Covid-19 testing or legislation on ascribing cause of death.

There are however important caveats to the excess mortality figures. Such numbers do not exclusively capture the increase in mortality that is directly caused by the presence of the novel virus. In addition, people may be less likely to visit hospital and therefore less likely to get treated for what are, in normal times curable diseases, thus tragically dying at a higher rate (Thornton 2020). Similarly, first response services may get overwhelmed and therefore be less able to respond to life threatening emergencies such as heart attacks and strokes, again causing higher than expected death rates (Oke and Heneghan 2020). Acting in the opposite direction, government responses to coronavirus such as lockdown, may reduce the number of deaths from other causes; transmission rates for other communicable diseases are likely to be suppressed while a reduction in travel reduces the mortality associated with traffic accidents (Alé-Chilet et al. 2020). It is therefore not a priori obvious whether excess mortality is positive or negative. Nonetheless, comparison of excess mortality with official Covid-19 deaths will provide a more informed picture of the overall impacts of the pandemic within and across countries.

Table 2 presents excess mortality data for the subset of OECD countries for which it is available. In general, the data are from The Economist (2020) but are supplemented for some countries by data from other sources.^5^ Baseline mortality is typically calculated as the mean number of deaths occurring in January-April 2015–2019.^6^ Excess deaths are calculated as the difference between the number of deaths observed in January-April 2020 and baseline mortality. The final column is the ratio of excess death to cumulative deaths at the end of April for each country, as reported by Our World in Data (Beltekian et al. 2020), calculated as:

1 $$Ratio = Excess\; deaths/Officially\;reported\;deaths$$

The heterogeneity that was present in the statistics of officially recorded Covid-19 deaths is also present in the excess mortality data. Some countries, such as Austria, Iceland and Portugal see only very marginal increases in death rates as compared with background death. There are countries which appear to do even better; Denmark, Finland, Germany, Israel and Norway all observing fewer deaths than expected. As discussed above, these negative excess death numbers could be the result of measures to combat Covid-19 reducing other-cause mortality, or from previous years used to calculate the baseline number of deaths being particularly bad. Indeed 2020 does seem to have been a year with relatively few deaths from influenza (Center for Disease Control 2020). At the other extreme, countries which appear worst hit based upon the officially recorded per capita death data are also those experiencing the highest percentage increase in mortality: Belgium, Spain and the UK all record deaths that are more than 15% higher than expected. Note that Italy too may well have been in this list, but the data for Italy is only available to 30th March, about the time the country experiences its peak daily mortality.

**Table 2:** Excess mortality data for the subset of OECD countries for which it is available

Country	Expected deaths (Jan-Apr average for 2015–2019)	Observed deaths (Jan-Apr 2020)	Excess deaths (Jan-Apr 2020)	Excess deaths per million	Excess deaths as a percentage of expected deaths (%)	Officially recorded Covi-19 deaths per million by end April 2020	Ratio of excess deaths to officially recorded Covid-19 deaths
Austria	30,600	31,140	530	59	1.74	66	0.89
Belgium	41,270	47,970	6700	578	16.22	647	0.89
Chile	33,660	35,940	2270	119	6.76	14	[8.42]
Denmark	19,910	19,440	− 460	− 80	− 2.33	85	− 0.95
Finland	19,810	19,580	− 230	− 42	− 1.16	44	− 0.94
France	209,570	227,820	18,250	280	8.71	357	0.78
Germany	298,950	290,930	− 8020	− 96	− 2.68	35	− 2.70
Iceland	820	830	7	21	0.85	29	0.70
Ireland	11,420	12,740	1320	268	11.59	241	1.11
Israel	12,950	12,360	− 590	− 68	− 4.55	23	− 2.92
Italy	156,870	172,640	15,770	261	10.05	192	1.36
Netherlands	53,740	61,070	7340	428	13.65	264	1.62
New Zealand	9910	10,490	580	120	5.84	4	[30.47]
Norway	15,180	14,390	− 800	− 147	− 5.24	36	− 4.13
Portugal	43,640	44,390	740	73	1.70	93	0.78
Spain	151,520	192,080	40,560	868	26.77	510	1.70
Sweden	31,940	33,400	1460	145	4.58	225	0.64
Switzerland	23,591	24,360	770	89	3.27	156	0.57
UK	220,360	264,950	44,590	657	20.23	394	1.67
USA	1,047,252	1,130,420	83,160	251	7.94	197	1.28

Calculations necessary to establish baseline expected mortality and the ratio of excess deaths to officially recorded deaths are discussed in text. Expected, observed and excess deaths presented to the nearest 10 (except Iceland). Excess and officially recorded deaths per million to the nearest death. Excess as a percentage of baseline and ratio to officially recorded are to 2 d.p

Turning to the ratio of excess deaths to officially reported deaths, again there appears considerable heterogeneity across countries, suggesting countries are indeed measuring the death toll from the pandemic by very different yard sticks. Generally, countries officially reporting high deaths tolls are also those which have the highest ratio of excess deaths to officially reported deaths. Indeed, Austria, Iceland and Portugal report more Covid-19 deaths than the excess deaths they experience. It is worth noting this is not to say that these countries are recording deaths as Covid-19 when they are not; rather it is entirely plausible the interventions to prevent Covid-19 in these countries have suppressed other deaths too. At the other extreme, some countries, notably the Netherlands, Spain and the UK, have ratios which imply upwards of 40% of Covid-19 deaths that are occurring are not being officially recorded. There are of course outliers to the overall pattern. Belgium, France and Sweden, have ratios below 1 despite having high per capita death tolls. Likewise, Chile and New Zealand have very high ratios, but these are almost certainly an artefact of them having so few Covid-19 deaths by the end of April, rather than because of under-reporting in each nation.

To recap, there are vast differences in the number of cases and deaths caused by coronavirus in different countries. This heterogeneity does not merely disappear when we account for potentially different reporting guidelines in each country; rather it may even be exacerbated. So what could be driving these patterns? While most countries chose to implement a relatively similar policy response, they did so at different times in their respective pandemics and some have been criticised for only belatedly imposing lockdown.^7^ There is some early correlative evidence that differences in current death tolls could be explained by lockdown date (Burn-Murdoch and Giles 2020) and we now move to consider this issue in greater detail.

## Policy Decisions and the Implied Price of Life

Our investigations of the potential impact of different approaches to reporting show the usefulness of an internationally agreed standard for assessing the impact of the pandemic. However, in the absence of such a standard we use national official estimates of Covid-19 mortality to understand the impact of lockdown policies. Data is supplied by the Our World in Data programme (Beltekian et al. 2020).

An initial task was to estimate the overall impact which policy responses could plausibly have had on Covid-19 mortality. To achieve this we undertook regression analysis examining the extent to which variation in Covid-19 deaths across all 37 OECD countries might be explained by socio-economic and demographic differences which no government could reasonably be expected to address during the timescale of a pandemic. A number of such exogenous determinants have already been highlighted in the literature. Of these one of the most clearly established mortality risk factors is a positive association with age; all other things considered, older sufferers are more likely to die from contracting Covid-19 than are younger people (Dowd et al. 2020). Therefore, across countries, populations which include a greater proportion of elderly people are likely to report higher death tolls. Similarly, those living in closer proximity to others may be more likely to pass on and contract the respiratory disease, hence variation in population density across nations may be a determinant of Covid-19 deaths (Rocklöv and Sjödin 2020). Beyond simple average population density, the degree to which populations are clustered in large urban centres may influence Covid-19-related mortality (Stier et al. 2020). Health outcomes might also differ because of within-country variation in wealth (Marmot 2005) which we capture in our regression by controlling for the Gini coefficient of income inequality for each country. Richer nations are likely better placed to limit the spread of pandemics (e.g. Hosseini et al. 2020), hence we use per capita GDP as a regressor to net-out cross-country differences owing to wealth. Finally, previous studies (e.g. Fraser et al. 2004) have highlighted that early detection may play a crucial role in halting virus spread, hence it seems plausible that countries which were exposed to Covid-19 earlier in the pandemic, and that therefore had less time to prepare, faced worse consequences. To account for this, we use the regressor “warning days”—the length of time (in days) between the WHO declaring that the Covid-19 outbreak was a “Public Health Emergency of International Concern” on 30th January 2020 and the country recording its 100th confirmed case (WHO 2020c).

The linear regression we use, details of which are presented alongside full results in Online Appendix 3, is deliberately simple and we are not claiming that the model necessarily captures causal relationships. However, even after including the list of exogenous factors which have been hypothesised to be major socio-economic and demographic drivers of cross-country variation in mortality rates, over 75% of the cross-country variation in Covid-19 mortality differences remains unexplained. Covid-19 deaths vary greatly across countries due to factors beyond socio-economics and demographics; the major remaining determinant is the policy responses implemented by national governments of which the most obvious difference is when different countries implemented lockdown.^8^

To investigate the impact of lockdown upon cross-country variation in Covid-19 mortality we calibrate country-specific SEIR models. SEIR models have a long history of development (Li and Muldowney 1995) with applications across a variety of infectious diseases including measles (Bolker 1993), HIV (Shaikhet and Korobeinikov 2016) and Ebola (Lekone and Finkenstadt 2006). More recently SEIR models have also been applied to Covid-19 (e.g. Annan 2020; Flaxman et al. 2020; Pei et al. 2020). However, as far as we are aware, ours is the first study to use the SEIR modelling framework to examine the effects of lockdown timing across multiple countries in the same study, and the first to combine these results with financial forecasts to obtain cross-country implied price of life estimates.

Price of life estimates derived in this paper are of critical importance given that government intervention has the ability to save life, yet trades-off against other goods. For example, closing schools is expected to reduce the transmission of infectious disease, hence decreasing the number of lives lost in a pandemic by imposing a human capital cost on today’s children (Viner et al. 2020). Likewise, there is evidence that the more stringent the government intervention to reduce the spread of coronavirus, the fewer lives that have been lost (Stojkoski et al. 2020). This too is not free: we all pay with restrictions on our basic freedoms. Beyond coronavirus, governments spend money and introduce legislation which imposes significant costs on society in a variety of sectors: healthcare (NICE 2012), road safety (DfT 2016), and safety at work legislation (HSE 2016). Governments also often have to consider multiple policy options for issues of environmental concern, be that considering pollution (Ackerman and Heinzerling 2002), climate change (Stern 2007) or biodiversity loss (Ellis et al. 2015). Here too, lives can be saved and lost as a consequences of policy decisions. Hence understanding how governments should value life is of critical concern. Indeed, a significant section of relevant policy documents is occupied by discussion of the value which a government should place on statistical life when evaluating policy (e.g. The Green Book; H.M. Treasury 2018).

In the case of coronavirus, there are already studies which aim to assess the economic value of particular policy interventions by reducing the number of lives lost. Hale et al. (2020) ask: how much of one year’s consumption would an individual be willing to forgo in order to reduce the mortality associated with Covid-19, suggesting the answer lies in the range one-quarter to one-half depending on exact mortality rates. Underpinned by assumptions about the rate of transmission and how policies may affect this, Greenstone and Nigam (2020) show the economic benefit of social distancing measures in the USA to be very substantial—about $8 trillion. Similarly, Thunström et al. (2020) use initial global estimates for the basic reproductive rate, and assume decreases to transmission from policy intervention from studies on Spanish flu, to go further. They conduct a cost–benefit analysis for similar measures, again in the USA, showing that the net benefits exceed $5.2 trillion. Gandjour (2020) and Holden and Preston (2020) conduct similar cost–benefit style analyses for Germany and Australia, respectively, both highlighting that lockdown comes out net positive. Here we ask a different but related question. Not whether lockdown makes economic sense, but rather what the timing of interventions reveal about the relative prices different governments place on their citizens’ lives. We focus on 9 countries with very different mortality rates and intervention timing—if there are discrepancies between countries for the price of life, they are most likely to be shown in this set of countries. In China, lockdowns were implemented on a province-by-province basis on very different dates. Therefore, at the country-level our GDP calculations would be incomparable with other nations. To overcome this challenge, we additionally parameterise an epidemiological model for Hubei, the province worst hit by the pandemic. We use the results from Hubei in our price of life calculations to maintain comparability across countries.

To be clear, the implied *price of life* should not be regarded as comparable to the *Value of a Statistical Life* (VSL).^9^ Specifically, VSL is a concept from normative economics—how much consumption *should* governments be willing to trade-off for an increase in the number of lives saved. This is a question which can be answered through stated-preference methods as has been done elsewhere (e.g. Alberini 2005; Carthy et al. 1999; Jones-Lee 1974). Rather, the implied *price of life* we calculate can be seen as an answer to the positive economics question of how governments actually *do* price lives saved in terms of consumption lost when making policy decisions.

### Calculating the Implied Price of Life

The key insight is that as the pandemic progressed governments continually had to decide when the moment was right to introduce a lockdown. Earlier lockdowns would save more lives, but likely impose greater immediate costs upon the economy. Likewise, delaying lockdown also delays the point at which a government becomes either morally or legally responsible for addressing the costs which such restrictions impose upon business. Therefore, *ex*-*ante* the expectation was that earlier lockdown meant greater financial cost. *Ex*-*post*, it seems governments may have been somewhat wrong to make that assumption as longer-term earlier lockdowns actually appear to be associated with shorter overall lockdown length, as is clear in Online Appendix 4, which in turn result in lower long-term economic costs (Balmford et al. 2020). Nonetheless, early imposition of lockdown imposed the certainty of cost, while a delay held out the possibility that the epidemic may turn out to be less severe than expected. Gambler governments chose to delay rather than act.

The chosen date of lockdown reveals a government’s preferences regarding the trade-off between avoided deaths and GDP losses.^10^ Relative to the chosen lockdown date, a later lockdown would have cost more lives, but reduced the financial impact. In its choice of lockdown date a government implicitly accepted the associated GDP loss rather than bear a greater death toll. Earlier lockdowns would have had the reverse effect; saving more lives but at a greater cost to the economy. In choosing not to enter lockdown earlier, the government rejected the higher financial cost of earlier lockdown in favour of more deaths. Hence, we are able to calculate both accepted and rejected prices for human lives: upper and lower bounds for the implied price of life in each country.^11^

A criticism of this method may be that decision makers at the time were unaware of the benefits of lockdown for public health. The evidence, however, points to the contrary. For example, it was reported in the print media at least as early as 7th March that the lockdown in Wuhan was showing signs of slowing the spread of coronavirus (Qin 2020). Within the UK there is evidence that scientific advisors notified the UK government of the benefits of lockdown two weeks prior to its imposition (Barlow 2020).^12^

Calculations of the implied price of life for each country require two data points. First, the differential effect on human lives lost from a marginal change in lockdown date. Second, the marginal effect on GDP from the same change in lockdown date.

### Modelling Deaths Across for Different Lockdown Dates

We use a compartmental epidemiological model to simulate the epidemic in each country and in particular to predict the outcomes of the counterfactual scenarios in which lockdown dates are changed. In this type of model, at any moment in time the population of a region or country is distributed between compartments according to disease status, and the function of the model is to describe (and predict) how the population flows between these compartments as the epidemic progresses. In the SEIR model which we are using, there are four compartments corresponding to Susceptible (i.e., not infected, but vulnerable to the disease), Exposed (a latent stage usually lasting a few days, where the victim has been infected but is not yet infectious), Infectious (at which point they can pass the disease on to others), and Removed (meaning they are no longer infectious and may be either recovered from the disease and immune, or else dead). In more complex models, the population may also be subdivided according to age and other factors, with each subdivision being compartmentalised according to disease status as previously described. This would allow for a more detailed representation of the structure of society and the progress of the epidemic as it spreads through the population, but such detail would greatly increase computational demands (especially for large ensembles of simulations as we are using here) and is not necessary for this work. For a full description of the model we are using, see Annan and Hargreaves (2020) and also House (2020) where the underlying model equations were originally presented. The flow of the population between the compartments depends on parameters which we estimate by fitting the model to observational data for each country. This model fitting process follows the standard Bayesian paradigm of defining prior distributions for uncertain parameters, running the model numerous times with parameters sampled from these priors, and calculating the likelihood on the basis of how well the model outputs match the specific observational data that we are using. This process (using a Markov Chain Monte Carlo approach) is described in detail in Annan and Hargreaves (2020). This approach requires around 15,000 model simulations for each experiment (i.e. country) and the results are represented by an ensemble of model simulations that samples our posterior probability distribution.

One critical parameter of the model, which has been widely discussed in the literature and media, is the reproductive number or R, which is the number of new cases that each infectious case generates in a fully susceptible population. If R is greater than 1, the epidemic initially exhibits exponential growth until it infects a sufficiently high proportion of the population that the remaining susceptible fraction substantially shrinks. If R is less than 1, the epidemic decays, again exponentially. In our estimation procedure, we assume that all uncertain model parameters are fixed in time apart from R, which is treated as piecewise constant. We consider three discrete periods within which R is constant. First, there is an initial period prior to “lockdown” controls being imposed by governments. A new, lower value for R is then assumed to apply during the period of strict controls, with a third value applying after the controls are significantly relaxed. Country specific lockdown dates that we use are detailed in Online Appendix 4. In reality, R and other model parameters are likely to vary somewhat during these periods but this piecewise constant approach has been widely used and captures the dominant features of the system (e.g. Flaxman et al. 2020).^13^

Due to serious limitations in the testing and reporting of case numbers, we rely exclusively on daily reported death numbers for the calibration of our model. Again, this is a common approach which is justified on the basis that the reporting of deaths is usually far more consistent and reliable than case numbers which depend strongly on testing capacity and policy. An alternative approach would be to use the number of excess death. While this may better reflect the number of deaths caused by Covid than reported death statistics, daily excess death data are not available. Moreover, the key results in the model are driven by changes in the rate of infection, hence even if death numbers in a particular country are underestimated due to systematic biases, this will not usually bias the estimates of model parameters. Therefore to calibrate the models we use daily reported deaths from Our World in Data up to 9th June (Beltekian et al. 2020), and later suggest how accounting for excess mortality would alter our estimates.

The prior estimate for R after the release of lockdown is taken to be N(1,0.2^2^) which represents our assumption that the policies are intended to be as open as possible while keeping the epidemic controlled. In many cases, there are insufficient data to constrain this prior estimate strongly, and therefore it plays a greater role in our results than the priors used in earlier phases of the epidemic. Estimates of all the R values, as well as our priors, are detailed in Online Appendix 5. Lockdown clearly reduces the infection rate across the board. Easing lockdown allows the infection rates to increase again.

**Fig. 2:**
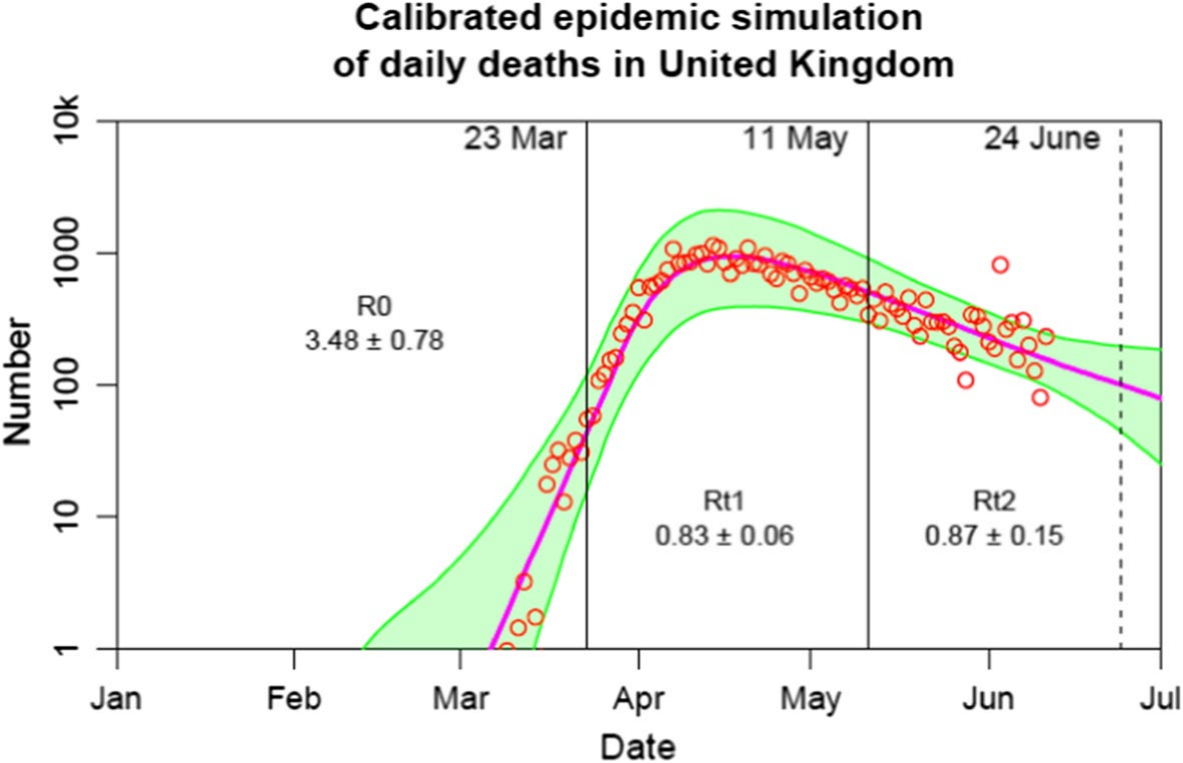
Observed and modelled deaths in the UK. Notes: The progression of the pandemic is divided into three time frames for each country: pre-lockdown (for the UK, before 23rd March), during lockdown (23rd March–11th May), and post lockdown (after 11th May). These time frames matter because the infection rate (R) changes as a result of imposing and subsequently easing lockdown. The posterior estimates for each period, and the 95% CIs are displayed on the graph

Figure 2 compares observed and modelled deaths in the UK, showing deaths on the (exponential) vertical axis over time. Modelled mortality (the solid line) closely matches the actually observed deaths (circles), illustrating that the modelling framework is flexible enough and the methodology sufficiently rigorous that the epidemiological model well replicates the observed patterns in the UK. Indeed, only on 3 days do observed deaths fall outside the 95% confidence interval (shaded area), and all such occurrences are in the post-lockdown period when the number of daily deaths is comparatively low. Similarly, close relationships are displayed for the other countries in the equivalent plots (Online Appendix 6), highlighting that the model well captures the country specific pandemic pathways.

In order to calculate the effects of changing the dates of lockdown, we use the fitted parameter values, and perform simulations in which the date of imposing lockdown is changed—either delayed or advanced by 3 days. We also explore advancing or delaying lockdown by 7 or 12 days, results of which are presented in Online Appendix 7. This approach is similar to that of others (e.g. Flaxman et al. 2020) in which the effects of policies have been analysed. Since we are using a single date to represent the net effect of multiple policies which were introduced across a period of several days, it would be more precise to interpret these scenarios as representing a change in the timing of all such policies by the given number of days. Likewise, we identify the impact of lockdown using within-country variation in the rate of infection. Therefore, to the extent that the stringency of policy interventions vary between countries, our simulations reflect the same country-specific set of policy interventions of the same stringency being implemented either earlier or later. That said, the lockdown is widely believed to be the most important of these measures (Flaxman et al. 2020) and so we consider our interpretation to be a reasonable approximation of the impacts of lockdown and variation therein. Differences in total mortality for each country dependent on date of lockdown are calculated to 24th June 2020. We also calculate the number of deaths that likely would have occurred were no lockdown implemented, again to the 24th June 2020. For illustrative purposes, the graph of predicted daily deaths for the UK under such a scenario is in Online Appendix 8.^14^ In all cases, no correction is made for the possibility that hospitals got overwhelmed, causing an increase in infection-fatality ratios. To the extent that such an outcome would have occurred, yet more lives would have been lost under the delayed- and no-lockdown scenarios.

### The Impact of Lockdown Decisions on Lives Lost

Table 3 highlights the likely impacts of lockdown policy. It is clear that the imposition of lockdown likely saved in excess of 14 million lives across the countries we examine. This overall analysis of lockdown is similar to that of Flaxman et al. (2020) and comparison of overlapping results shows that they are in most cases strikingly similar.^15^ However, we caution against over-interpreting the result: it is likely that even without a formal lockdown, people would have socially distanced and engaged in other behaviours to limit Covid-19 deaths. Nevertheless, earlier governmental action would have saved a large numbers of lives, particularly in countries such as the UK and US who acted relatively late. Pre-lockdown reproduction rates are substantially greater than one, hence across all countries, longer delays result in exponentially greater losses of life.

**Table 3:** The human impact of imposing lockdown, and how that would have varied by earlier or later intervention

Country	Lives saved by lockdown Mean [95% CIs]	Additional lives that would have been saved by imposing lockdown 3 days earlier Mean [95% CIs]	Additional lives that would have been lost imposing lockdown 3 days later Mean [95% CIs]
Belgium	71,000 [43,000; 104,000]	4600 [2000; 8200]	7700 [2700; 14,700]
China	10,112,000 [5,277,000; 14,139,000]	N/A	N/A
China (Hubei)	67,000 [41,000; 90,000]	2200 [500; 5400]	4000 [700; 10,700]
Denmark	35,000 [2000; 54,000]	300 [100; 600]	500 [100; 1400]
Germany	539,000 [375,000; 717,000]	4000 [1900; 7600]	7900 [3100; 17,500]
Italy	378,000 [207,000; 570,000]	18,100 [6700; 33,000]	29,100 [10,000; 57,600]
Korea	276,000 [0; 455,000]	105 [12; 240]	182 [12; 509]
New Zealand	30,000 [5,000; 45,000]	37 [10; 70]	72 [13; 169]
United Kingdom	424,000 [247,000; 607,000]	20,000 [8800; 38,000]	32,000 [13,100; 62,000]
United States	2283,000 [1382,000; 3121,000]	51,100 [30,000; 84,000]	90,000 [44,000; 152,000]

Lives saved by a lockdown are rounded to the nearest thousand deaths. Additional lives saved/lost to the nearest hundred, other than for Korea and New Zealand which are to the nearest death; their early intervention means marginal differences to lockdown date make relatively little difference to the number of deaths

### Economic and Financial Consequences of Lockdown

The previous sub-section presented clear evidence that the choice of when to impose lockdown drastically affects the likely number of deaths. Moreover, there is significant heterogeneity across countries in the number of lives that would have been saved had lockdown been implemented just 3 days earlier or later. How does this heterogeneity translate into the implied price of life across countries?

To assess the price of life we require estimates of the financial cost of lockdown on GDP. We first assume that the full cost of any extension to the length of lockdown is felt in the year 2020. Therefore, we estimates the cost to GDP by comparing the last IMF forecasts of national GDP in 2020 prior to the pandemic (from October 2019; IMF 2019) with their most recent forecast for 2020 (April 2020, IMF 2020b).^16^

Further assumptions are needed to understand the cost of a marginal extension to lockdown. The first is the relationship between lockdown length and cost to GDP. In line with the best available evidence, from studies in the US (Walmsley et al. 2020) and thirty pan-global countries (with a focus on European nations, Fernandes 2020), length of lockdown appears to be directly proportional to the percentage GDP loss. Of course, not all of the GDP loss associated with an extended lockdown is the result of the policy decision alone: progression of the pandemic sufficient to warrant a lockdown (extension) would reduce GDP outlook anyway and there is good evidence that people were changing their behaviours to enact social distancing in advance of direct regulations (Gupta et al. 2020). Moreover, it is not just the domestic pandemic which causes GDP losses—some is also driven by the state of the virus in other nations owing to trade (Mandel and Veetil 2020). Hence we must also make an assumption about how much of the loss in GDP in any given country is the result of the lockdown policy, rather than other factors associated with the ongoing pandemic. Andersen et al. (2020), Chronopoulos et al. (2020) and Goldsztejn et al. (2020) have all teased apart the effects of lockdown policy from the wider pandemic. All three suggest that the GDP loss caused by lockdown policy is approximately 15% of the total GDP loss experienced by each country.^17^ We note of course that there are reasons to believe this figure could be an over- or under-estimate of the proportion of cost attributable to the lockdown policy, and that this could also vary somewhat by country given that lockdown policy may have different impacts on different industries.^18^ Nonetheless, we see the 0.15 estimate as offering a reasonable ball-park figure, and so adjust predicted GDP losses as per Eq. 2:

2 $$\Delta GDP_{ij} = \left( {\frac{{\Delta Lockdown \;length_{i} }}{{Actual \;lockdown\; length_{j} }}} \right) \times IMF\; forecast\; GDP\; loss_{j} \times 0.15$$

Equation 2 states that the GDP loss caused by changing the length of lockdown by some amount (either 3, 7 or 12 days; denoted $$i$$), in country $$j$$, is calculated as the relative change in lockdown length, multiplied by the predicted change in GDP as forecast by the IMF, and the proportion of the loss attributable to the policy decision ($$0.15$$). We adopt the IMF metric for measuring GDP in terms of Purchasing Power Parity International dollars (PPP$) which is held constant such that it is equal to the US dollar. For Hubei, we use the same formula as above, however the IMF only publishes estimates GDP forecasts at the national level. Therefore we partition the effect for Hubei alone by multiplying by the proportion of China’s GDP which Hubei makes up (0.04,651).^19^ The necessary data, and calculated GDP outcomes, are presented in Online Appendix 8.

It is worth highlighting two further implicit assumptions. First, we assume all of the GDP loss a country experiences occurs during the lockdown period. Clearly, countries’ economies were already contracting pre-lockdown, and likely will take a long time to return to normal functioning post-easement. However, our assumption ensures that the implied price of life we calculate is an upper bound. Second, we assume that the date on which lockdown is eased is independent of the date on which lockdown was imposed. This is an open empirical question as it may be that earlier lockdowns halt the spread of the virus quicker, allowing an earlier end to lockdown. If earlier lockdowns result in earlier release this would lower the overall financial burden of lockdown. Hence, again our assumption tends towards an upper bound estimate on the price of life. The additional assumption made for Hubei may underestimate the price of life there: the contraction in China’s GDP is likely most keenly felt in Hubei, the worst hit province. Our estimates of price of life would increase if we adjusted for this.

Aside from the caveat with respect to China, while our assumptions influence *absolute* estimates of the price of life, the only variables affecting the *relative* prices across countries are: (1) the number of lives a change in the length of lockdown would save; (2) the original length of lockdown in a country; and (3) a country’s GDP. These key variables are not assumed. To underscore the point, our assumptions cannot substantially influence the implied relative price of life across countries.

### Cross-Country Estimates of the Price of Life

To calculate the implied price of life from a change in the length of lockdown of a set number of days, $i$, for country, $j$, we link the predicted change in GDP to the change in number of lives lost as in Eq. 3:

3 $$Implied\;price\;of\;life_{ij} = \Delta GDP_{ij} /\Delta Lives\;lost_{ij}$$

Our primary focus is for the most marginal change in length of lockdown we calculate: imposing lockdown either 3 days earlier or later than its actual date. Results for different changes in lockdown date, of 7 and 12 days, are presented in Appendices 9 and 10. These show that relative patterns remain unchanged. Table 3 showed that the exponential growth in infections means more lives are lost from a delay, than would be saved by shifting lockdown earlier by the same number of days. In contrast the modelled impact on GDP from moving the lockdown date by a fixed number of days is exactly the same; the only difference is in the sign (earlier lockdowns are a cost to GDP, later lockdowns a benefit). Hence, the implied price of life is higher for moving lockdown earlier as opposed to later. Moreover, as explained previously, by choosing not to impose lockdowns 3 days earlier governments *rejected* saving more lives when the price was relatively high. Similar logic reveals them to have *accepted* the implied price of life from a delay; they would rather bear the cost in terms of GDP than as further human lives lost. Results from these analysis are presented in Table 4.

**Table 4:** Implied price of life in different countries (PPP$)

Country	Accepted price of life Mean [95% CIs]	Rejected price of life Mean [95% CIs]
Belgium	55,000 [29,000; 155,000]	93,000 [52,000; 211,000]
China (Hubei)	108,000 [41,000; 597,000]	202,000 [80,000; 950,000]
Denmark	807,000 [293,000; 6446,000]	1515,000 [657,000; 7082,000]
Germany	525,000 [238,000; 1336,000]	1035,000 [547,000; 2218,000]
Italy	59,000 [30,000; 172,000]	95,000 [52,000; 257,000]
Korea	6682,000 [2389,000; 101,341,000]	11,563,000 [5063,000; 102,135,000]
New Zealand	3450,000 [1470,000; 19,106,000]	6762,000 [3548,000; 24,100,000]
United Kingdom	67,000 [35,000; 166,000]	108,000 [57,000; 248,000]
United States	87,000 [51,000; 177,000]	152,000 [93,000; 258,000]

These results use changes in lives lost and financial estimates associated with a 3 day perturbation in the lockdown date. Accepted price of life calculated as the trade-off between GDP and life imagining lockdown had been imposed 3 days later; rejected price of life as if lockdown had been imposed 3 days earlier. Prices to the nearest thousand

Obviously, estimates for prices countries were willing to pay (accepted) are lower than estimates for the prices countries rejected. In almost all cases the estimates of the price of life are below thresholds typically used to estimate the VSL in cost–benefit analyses. Hence, *ex*-*post*, it is highly likely lockdown enhance social welfare.^20^ As with progression of the pandemic, there is huge heterogeneity in the price of life across countries. Comparing across countries those who pursued an early lockdown strategy reveal they are willing to pay a high price to save their citizen’s lives, only rejecting prices above $1,000,000. The highest implied prices are in Korea (> $11,000,000) and New Zealand (> $6,000,000), both countries who acted swiftly to suppress the pandemic.^21^ However, those countries which imposed lockdown relatively late-on in their respective pandemics were clearly only willing to pay far less to protect lives. Belgium, Italy and the UK reject prices of life around $100,000.

Clearly, delayed action in the face of exponential growth cost lives, and implied low price of life in those countries imposed lockdowns relatively late in the pandemic. Two comparisons make this cross-country variation in the implied price of life particularly clear. First, the accepted price of life in China ($108,000) is about 25% higher than that for an American ($87,000). This is despite our methods meaning the calculated price of life for China is likely an underestimate.^22^ Second, compare the acceptable price of life in Germany ($525,000) with that in the UK ($67,000). The price of life for a German is nearly an order of magnitude greater than that for a British citizen. That vast difference is despite the two countries being very similar in terms of GDP per capita. These *relative* implied price of life comparisons are particularly pertinent. Our methodology uses *ex*-*post* estimates of the number of lives saved to infer what government policy implies for the price of life. Yet, these governments were clearly making the decisions *ex*-*ante*. Nonetheless, these governments were making lockdown decisions at around the same time (except Hubei which was far earlier), with nearly identical information sets. Thus any differences in relative estimates would hold true even if the pandemic had proved to be far less deadly than it actually is.

Moreover, this heterogeneity in the price of life is not explained by different values for life. Indeed, the implied prices are often far lower than official VSL estimates—seemingly, cash flowing through the market is worth much more than value passing through wellbeing, at least to some countries. The low rejected prices also imply that very few Quality Adjusted Life Years (QALYs) are assumed to be saved by governments in reducing Covid-19-related mortality; otherwise delays to lockdown seem nonsensical. For reference, in the UK the National Institute for Health and Clinical Excellence views a QALY costing between £20,000 and £30,000 as good value (NICE 2012).

As we mentioned when discussing Table 2, those countries with high reported Covid deaths, tend to be countries with high ratios of excess mortality to reported death, i.e. there is substantial under-reporting. To examine the extent to which our estimates change when we account for this under-reporting, we focus on the set of countries for which we have reliable estimates of that ratio, and where under-reporting appears prevalent. These countries are: Italy, the UK and the USA. The estimates reported in Table 5 are calculated by dividing the estimates of the price of life by the ratio of excess mortality to reported deaths (from Table 2). The intuition behind this is that our estimates of lives saved by lockdowns (used in Table 4) are based upon reported death data, and hence should be scaled upwards by the degree of under-reporting of deaths. Implicit in this correction is the assumption that the ratio of excess death to reported death is constant within a country throughout the pandemic. It is possible that the ratio declines during the tail of the pandemic when Covid cases and deaths are less common, and tests more available. Nonetheless, our correction offers what is currently the most comparable cross-country figure.

**Table 5:** Implied price of life in different countries after correcting for under-reporting (PPP$)

Country	Accepted price of life Mean [95% CIs]	Rejected price of life Mean [95% CIs]
Italy	43,000 [22,000; 126,000]	70,000 [38,000; 189,000]
United Kingdom	40,000 [21,000; 100,000]	65,000 [34,000; 149,000]
United States	68,000 [40,000; 138,000]	119,000 [73,000; 202,000]

As described in text, these figures are calculated by dividing the price of life estimates in Table 4 by the country-specific estimate of under-reporting (ratio of excess death to reported Covid death) in Table 2. Prices to the nearest thousand

Table 5 shows that for those countries which under-report Covid-19 deaths, implied price of life is substantially reduced, highlighting once again that earlier lockdowns would have increased social welfare tremendously. For example, in the UK, the country for which we estimate a relatively high rate of under-reporting of Covid-19 deaths, the adjusted rejected price of life is just $65,000 (equivalent to just over £50,000). The accepted price of life is lower still, at $40,000 (£32,000).

## Concluding Remarks

This study has begun to disentangle the extent to which cross-country comparisons of responses to Covid-19 are valid despite difficulties caused by both exogenous factors and differences in testing rates and the recording of cases and deaths. The results presented in this paper suggest that policy interventions may well explain the majority of cross-country variation in officially reported Covid-19 deaths.

For some countries, deficiencies in official approaches to the recording of Covid-19 mortality mean that estimates based upon deviation of overall deaths away from the seasonally expected norm may provide a more accurate depiction of fatalities caused by the pandemic. Such ‘excess death’ estimates suggest that in some, highly impacted, countries the actual number of Covid-19 deaths may considerably higher than indicated in official statistics. For example, within the UK it seems that more than a third of Covid-19 deaths may have gone unrecorded. Where under-recording is prevalent, then the number of lives lost by delayed intervention (as well as those saved relative to even further delay) is likely to be substantially higher than estimated in this paper. Any such under (over) estimation of true deaths would result in an over (under) estimation of the price of life implicit in lockdown decisions.

Careful consideration of cross-country differences is required if we are to glean the important natural experiment evidence afforded by countries implementing different policy approaches to the pandemic. The results presented in this paper highlight that well-designed policy can save life. While the economic burden of lockdown is large, comparison with prior decision criteria suggest that such policies generate net benefits for society.

## Electronic supplementary material

Below is the link to the electronic supplementary material.

Supplementary material 1 (DOCX 491 kb)

## Footnotes

1 Kurmann et al. (2020) note that small business employment contracted by 60% (over 18.2 million) between mid-February and mid-April 2020 since when over 9 million had been rehired to the end of June 2020.

2 *Source*: Worldometer, https://www.worldometers.info/coronavirus/country/us/ accessed 4th July 2020.

3 The figure is a redrawing of one which was displayed daily at the UK press briefing from 30th March 2020 until being left out of daily briefings from 10th May 2020 onwards. Speeches by the Prime Minister on Covid-19 had been conducted before then (for example on the 9th and 12th March) but they only became a daily occurrence with a relatively standardised format from 16th March onwards. Slides from these briefings are available here: https://www.gov.uk/government/collections/slides-and-datasets-to-accompany-coronavirus-press-conferences.

4 The verbal explanation which accompanied the presentation of Fig. 1 each day is gained by re-watching the recordings hosted by the government’s official YouTube channel: https://www.youtube.com/user/Number10gov/videos. As discussed in text, transcripts of the relevant section are provided in Online Appendix 1. AntConc software (Anthony 2019) was used to search for particular phrases. These phrases, and the daily counts for each, are given in Online Appendix 2.

5 The other data sources used for particular countries are: Austria - http://www.statistik.at/web_de/statistiken/menschen_und_gesellschaft/bevoelkerung/gestorbene/index.html); Belgium—https://epistat.wiv-isp.be/momo/; Finland - https://pxnet2.stat.fi/PXWeb/pxweb/en/Kokeelliset_tilastot/Kokeelliset_tilastot__vamuu_koke/statfin_vamuu_pxt_12ng.px/; Iceland—https://hagstofa.is/utgafur/tilraunatolfraedi/danir-tt/; Ireland (note these are death registrations rather than government figures)—https://rip.ie/Deathnotices/All; Israel—https://www.health.gov.il/UnitsOffice/HD/PH/epidemiology/Pages/epidemiology_report.aspx?WPID=WPQ7&PN=6; New Zealand—https://www.newsroom.co.nz/2020/05/05/1,157,173/are-there-hidden-covid-19-deaths-in-nzs-statistics; Spain (importantly accessed on 11th June, after there was a major addition to the figures)—https://www.scb.se/contentassets/edc2b33f85ad415d8e7909002253ed84/2020-04-09%E2%80%94preliminar-statistik-over-doda-inkl-eng.xlsx; USA—https://data.cdc.gov/NCHS/Excess-Deaths-Associated-with-COVID-19/xkkf-xrst. Even among the countries for which data is available, mortality data are only available for a few months of the year, generally at least to the end of April, hence the focus January–April 2020 deaths. Data tend to be aggregated to the week level, hence the exact endpoint is rarely 30th April 2020. Rather, the last day used in 2020 is determined by the data availability, and chosen to be as close as possible to 30th April. In all cases, we compare like-for-like, such that the baseline deaths are recorded over the same time period. Likewise, the cumulative death toll we use to calculate the ratio of excess to reported death is that which was officially reported on the last day of the 2020 mortality data we use for each country.

6 For some countries data availability means this is not possible. For Austria, Belgium and Germany it is 2016–2019; Iceland and USA use 2017–2019; for Spain baseline deaths are modelled by MoMo.

7 For example, the UK has been criticised for delaying lockdown (Scally et al. 2020).

8 Indeed, as is reported in Balmford et al. (2020) a similar regression, dropping all covariates and only including the delay to enacting lockdown explains nearly 40% of total variation. The effect is robust to including the covariates we specify here but overall r^2^ is only marginally increased, and the adjusted r^2^ is actually lower than with the lockdown delay alone.

9 The distinction between price and value in the context of life is much the same as that in the diamond-water paradox highlighted by Adam Smith (1776).

10 That such a trade-off is inevitable and in principal morally defensible is not questioned, indeed it follows logically from the VSL. Increasing economic costs impact upon human welfare. An approach which says that every life is of infinite value would impose infinite costs upon the economy, resulting in far greater losses of human wellbeing (and almost certainly life) than acting in a way which imposes an implicit and non-infinite price on life. It is the cross-country comparison of that implicit price which is examined here.

11 Our focus on GDP reflects both the ubiquity of this measure and a lack of available, robust, economic estimates of the wider welfare impacts of lockdown. To better understand some of those wider costs, we direct the interested reader to: Branley-Bell and Talbot, 2020; Burki, 2020; Cash and Patel, 2020; Chaix et al., 2020; McLay, 2020; Pancani et al., 2020; Pinto and Jones, 2020; Sud et al., 2020; Van Lancker and Parolin, 2020. While driven out of necessity, we think that a focus solely on GDP is also justified. Our interest is in the relative price of life across country. Even accounting for the external costs, the relative pattern for price of life would remain; it could only be eroded if these external costs are disproportionately larger for countries with lower GDP-based price-of-life estimates.

12 Indeed Grant Shapps, a UK Government minister, was questioned on 16th March 2020, a full week before the UK entered lockdown, regarding why the UK was following the example of other countries in implementing a lockdown given evidence that such a response seemed to work. A summary of the interview is available on the Sky website here: https://news.sky.com/video/coronavirus-uk-approach-entirely-science-led-grant-shapps-11958199. There is also a video of the interview on the Sky Facebook channel here: https://www.facebook.com/watch/?v=230181738109777.

13 Note that any changes in R between time periods captures changes due both to lockdown itself, and any other policy changes to the extent they co-occur with changes in lockdown legislation. That said, Flaxman et al (2020) make clearly show that the vast majority of policy impact on R is driven by the decision to enter lockdown.

14 The graphs are similar for all other countries, and hence not displayed here.

15 Note however that our estimates for lives saved in Belgium and Italy are somewhat more conservative than those of Flaxman et al (2020), owing to our slightly lower infection-fatality ratio.

16 IMF forecasts for GDP are in line with those provided by other experts. For example, recent IMF predictions for the UK represents the median from a range of other forecasters (Resolution Foundation 2020 as cited in Richiardi et al. 2020). Note that we assume that the IMF’s April 2020 GDP forecast reflects an accurate expectation of the length of lockdown imposed in each country.

17 Andersen et al use data from individual-level transaction data either side of the border between Denmark, which imposed a lockdown, and Sweden, which did not. Denmark saw transactions reduce 29% in the immediate aftermath of lockdown imposition compared to Sweden’s 25% reduction. This suggests that 13.8% of the GDP loss Denmark experienced is caused by the lockdown rather than mere pandemic progression. Chronopolous et al present evidence from either side of the UK lockdown, again using individual-consumer-level transaction data. This suggests a similar proportion of the overall cost is attributable to the lockdown policy: spending drops by 15.2% in the week following lockdown (week beginning 23rd March 2020) relative to the previous period. Goldsztejn et al conduct a modelling exercise linking economic data to an SEIR model for the UK again. This suggests that lockdown accounts for 17% of the overall economic downturn.

18 As more accurate estimates of this key parameter become available, we would encourage the interested reader to replicate our calculations but with an updated estimate of the proportion of GDP loss attributable to lockdown policy to provide more accurate estimates of the price of life.

19 This is calculated from estimates of 2019 provincial GDP published by the National Bureau of Statistics China, downloadable here: http://data.stats.gov.cn/english/easyquery.htm?cn=E0103 [Accessed 12th June 2020].

20 Ideally we would assess all of the consequences of interventions (e.g. the mental health costs of lockdown) before making such an assertion. However, the difference between VSL values and our price of life estimates suggest that our statement is defensible (certainly for those countries where the latter measures are particularly low).

21 Moreover, Vietnam would have been included in the modelling exercise, but we were unable to robustly parameterise our epidemiological models as so few cases (let alone deaths, of which there have been none) have occurred.

22 This is true to the extent to which officially reported Covid-19 deaths in China are accurate. If officially reported deaths are far lower than the number of deaths which have actually occurred, this figure may well be an overestimate of the price of life in China. We have not found data from China on excess mortality and so cannot speculate on the degree to which mortality data are accurate.
