# Supplementary material for: Cross-Country Comparisons of Covid-19: Policy, Politics and the Price of Life
Source: Environ Resour Econ (Dordr). 2020 Aug 4;76(4):525–51. doi: 10.1007/s10640-020-00466-5 (PMC7400753; doi:10.1007/s10640-020-00466-5)
Supplement: Supplementary file 1 — Supplementary material 1 (DOCX 491 kb) [file 10640_2020_466_MOESM1_ESM.docx]

**Online Appendices**

**Appendix 1:** Verbatim transcripts from UK Government press briefings on the progression of the pandemic in the UK referred to in our discussion of Figure 1 in the main text

*30^th^ March*

All of this is about preventing deaths and preventing the NHS becoming overwhelmed in the intensive care units with ventilators, and on this graph it tracks the deaths that have occurred globally across some of the countries, not all of the countries obviously, and it shows that there is a pattern of increasing deaths which you expect to reach a plateau, to come down eventually and you can see in the UK here which is in the purple line, we are tracking roughly along the same path as France, I've said before we're behind Italy in terms of the curve, you can see that Spain has a higher number than Italy at the moment - in terms of its trajectory, not in terms of the total number, but the direction in which it's going. The UK is tracking alongside France in this.

*31^st^ March*

As the next chart shows: hospitalisation. Unfortunately, some people who are hospitalised do die, every death is tragic and we absolutely need to avoid as many deaths as possible by all playing our part. So what we want to see over time is a reduction in the number of deaths and you can see here a comparison of deaths in different countries and you can see maybe in the very light line in the middle, that China over time has flattened that particular line, in other words the number of deaths have reduced. And so if we reduce the number of infections, we will reduce the number of hospitalisations and we will reduce the number of deaths.

*1^st^ April*

And this is what it's all about, it is about saving lives. This slide shows the global death comparisons. We need to save lives. As things stand, it has not been as severe here as in France, and we are just tucked in under the USA and obviously Italy on a different trajectory but - and Spain and the United States, as we said, but there is no reason to be complacent. Stay at home, protect the NHS, save lives.

*2^nd^ April*

And that finally of course that translates unfortunately and with great sadness into deaths, and we are seeing the number of deaths increase and as I've said unfortunately I think that will continue to be the case, we will still see those high numbers of deaths for a few weeks yet, until the benefit of all the actions that everybody in the country is taking will start to bear fruit and will start to impact into a reduction in deaths.

*3^rd^ April*

The final slide shows the comparison of the UK in relation to various other countries and the index is set at the first day in each country when 50 deaths were reported and you can see from this slide that it is a global battle and that the UK is in the same situation in terms of the curve and the shape of the curve as many many other countries around the world.

*4^th^ April*

And of course finally as the next chart shows unfortunately and with great sadness the number of deaths continues to be high and as I and others have said unfortunately that is likely to continue over the next week or two until we get on top and stop this virus and by doing that we will see a reduction in the number of deaths.

*5^th^ April*

And again this gives an indication of the global death comparison. Now, again, as many of my colleagues have said before this tries to give a direct comparison even though some of the international comparative data is difficult to interpret, but I think it’s important that we continue to look and where necessary and where possible to learn from other countries. The UK in this slide is in blue, in the centre of that grouping, and these are the deaths which have all been calculated from a 0 day, that have been brought back to when the first 50 deaths were reported in the country. Now for the UK these are covid-associated deaths, they are all sad events, they will not all be a death as a result of covid, but certainly we need to be sure that we are counting everything appropriately and being very clear and transparent on that, so we will continue to track and monitor this and learn from other countries where we can.

*6^th^ April*

The final set of numbers to look at today is the global comparisons of these very sad deaths from covid, and this is the most lagged data. So these are data that show the data reporting of deaths and that can take a very long time after someone’s died and be rather variable how long that takes in different countries and we will expect - even if the number of people being admitted to hospitals and ITU units does start to stabilise – sadly we do expect this number to keep on rising because of course it takes time after have been admitted to hospital for them either to get better and very sadly to die, and then of course further takes time for those deaths to be reported.

*7^th^ April*

These are the graphs showing the number of deaths across different countries and you can see that despite a little uptake in the numbers today and the numbers do bounce around, the UK is on track; we are roughly a couple of weeks behind France, a few weeks behind Italy in terms of the numbers, but you can see that broadly things across Europe move in the same direction. And we would expect the number of deaths to lag after the ICU cases by a couple of weeks, so we should expect these to start coming down in 2 or 3 weeks’ time.

*8^th^ April*

So finally I have data on the sad statistics of people who have died from covid, and this is an international comparison across different countries, showing how that number accumulates in different countries and the UK is the dark blue line somewhere in the middle there. This data has long reporting lags and even after the number of people in critical care stabilises or even maybe begins to fall, this number will rise because sometimes deaths are reported many many days or even a week or so after sadly somebody has died. So we expect this number to keep rising even after the curve has flattened.

*9^th^ April*

Unfortunately, sadly, there are deaths from this disease. And those continue to rise and you can see here the increase in rise in the UK and in other countries. This will not change for a few weeks, because the deaths come after the other illnesses, the early illness, the intensive care and then some patients, unfortunately, die. And that will continue for a few weeks we need to see this begin to go down as well, it should follow the others.

*10^th^ April*

If you look at the global deaths comparison on this slide – again, as last week, these are synchronised so that day nought is the day at which each country reported 50 deaths. And you can see that again there is this broad similar trajectory across many countries that report data, with one or two outliers, and the UK continues to be broadly in line with that is happening in other countries. It’s still a dangerous situation we have to keep taking measures to bring this under control.

*11^th^ April*

And then in the next chart unfortunately as I and others have said before we are still seeing sadly high numbers of deaths. That will be the very final thing that will change and start to decrease but we are confident that if everybody applies- follows the instructions, complies and follows the instructions we've all been given, that will begin to translate in the next week into a reduction in the daily deaths.

*12^th^ April*

On the final slide we have the global death comparisons and here we can see where the UK is, it's somewhere in the middle, it's tracking France, it's looking close to the Italian trend at the moment but this is still early days, we would stress, in understanding deaths and we can see where other countries have had increases in their deaths at the moment.

*13^th^ April*

This is the slide of deaths and again it doesn't carry all deaths in this, it looks at the deaths in hospitals and the reason it looks at the deaths in hospital is that's the international standard on which everyone else is doing in terms of reporting deaths. There are of course unfortunately many deaths that also occur outside hospital. We're tracking behind Italy, we are following the same sort of path. What do I expect to see happen? I think this week's difficult, I think this week we're going to see a further increase; thereafter we should see a plateau as the effects of the social distancing come through. That plateau may last for some time and then begin to decrease, that's what we'd expect to see in this very unfortunate death curve.

*14^th^ April*

And then on the next slide you will see the number of deaths in the UK and as you've heard that is continuing to rise. This is the number that will reduce last, unfortunately with sadness this is the one that will take longest to change but those benefits from social distancing will eventually translate into a reduction in the number of daily deaths.

*15^th^ April*

The final slide is comparing the sad deaths that we've had in our country with those in other countries. These curves are all lined up so that they all start on the day in which each of those countries first had 50 deaths and you can see the line in dark blue in the middle there is the United Kingdom's trajectory: still climbing, we'd always expected that to still be climbing, even though the number of new cases is starting to flatten off and that is because these are very lagged data because of course sadly it takes a while for people first of all to succumb to this disease and then secondly for those cases to be reported. The United Kingdom data there are from Public Health England and from our devolved administrations and we're watching and hoping that those two will soon start to follow a flatter trajectory.

*16^th^ April*

Sadly of course there are people who've died from this awful virus. I expect the deaths to reach a plateau and to stay up a bit for a little while and then to decrease thereafter. And what you can see on this slide is both the deaths in hospital and in the all settings from the Office for National Statistics' data, so the total picture can be seen.

*17^th^ April*

Unfortunately, as the Secretary of State has said, deaths continue to occur from this disease. 847 in the last 24 hours. This is obviously a tragedy. I expect unfortunately this number to continue at a plateau for a little while and then to start coming down after that.

*18^th^ April*

As we've often said unfortunately a reduction in the number of deaths will be the last thing that will change, but if we continue to comply with instructions it will change. Here we are now presenting deaths data for hospitals, the hospital data comes rapidly, we can get that very quickly and therefore it's right that that is published immediately, but we can also collect deaths- data on deaths in the community and now you will see a second line with some lag on it, which we are working hard to decrease the lag, looking at death in all settings.

*19^th^ April*

And then the last slide here is our global deaths comparison, again this is one which, as a public health specialist, epidemiologist, is one which we need to reflect back on many months ahead in a actual fact because of the difference in the way which different countries and different systems collect data, but we want to be able to show you what is happening in the UK and how that relates as far as we possibly can to other countries and you can see there are two lines on this one. The UK hospitals only data, this is very robust data which is readily accessible and you can see that blue line progressing on the right but we like to think that it's starting to even out a little bit. And then you will see to the left of the slide a UK all settings data line which is reflecting the fact that we have a lag if you like between our community death reporting and this endeavour to try and display in line with some other countries recording mechanisms how the UK is managing to that extent.

*20^th^ April*

Finally we get to the slide comparing how many people have died in the UK in comparison with other countries. We have two different ways of keeping track of that here, we have the most- the very up-to-date data which goes right to yesterday and that is UK but only counts deaths that occurred sadly to people who are in hospital and those are deaths by the data registration, and then further back on that slide you can see data from the UK for all settings and that data takes longer to put together and present so that's why on this slide that data is only a shorter data run.

*21^st^ April*

I will show you a slide I've shown you before about global death comparisons. Again, as I've said before, the slide is indexed on time-point zero when a country declares its first 50 cumulative deaths. Now, for almost all of the countries on this slide the reporting as in the UK has been for hospital deaths. And you can see there that there are broadly three patterns: the United States on its own, European countries other than Germany in the middle of the pack and some outliers who've had very low rates of death. On the slide if you look carefully you will see a curve for UK hospitals only and a UK all settings which is- lags further behind, which includes all of the deaths reported to the ONS, but you will see that they are on the same trajectory within the same framing of those middle countries. These are important comparisons, but what will be really important in the future, and we do not yet have those data, are international comparisons of excess deaths, particularly across Europe where a pretty standardised methodology is used for counting excess deaths but those data take time to assemble and I don't have them for you today.

*22^nd^ April*

And this is a slide we use just really to track the trajectory between different countries I should be clear that trying to compare different countries is in this kind of data- is notoriously difficult but it does show the trajectories between the different countries with the UK using hospital data which is our most steady source of data over that time. Next slide please. This is just one slide we thought we would add in in addition because I think it makes a point which I think is important for people fully to understand. And what you can see here, this is the 7-day rolling average for deaths sadly in several countries including the UK. And the reason I though it sensible to put this in was for people to see that even in those countries which started their epidemic curve earlier than the UK and which are still ahead, the downward slope from the point where we change is a relatively slow one and we should anticipate the same situation in the UK, we should not expect this to be a sudden fall away of cases.

*23^rd^ April – slide is present in presentation material but not discussed in briefing*

*24^th^ April*

And then finally this tries to give an indication, it's very difficult because of the difficult comparisons of death data between countries. But to indicate how the UK is doing in relation to other parts of the world. The far right blue death line which you can see marked as "UK (hospitals only)" indicates the data that we've just seen on the preceding slide. And then increasingly we are trying to provide a much broader indication to ensure we are capturing death which is not just in hospital, but that we know will be happening in communities, to make sure that we clear how we are managing the disease and to make sure that we are looking after those who are most vulnerable.

*25^th^ April*

The global death comparison, which again we show and again this shows UK hospitals only but also, with a lag on it, it also shows deaths in UK all settings. So this includes deaths out of hospitals, but as we've said on a number of occasions there is more of a lag in the collection of that data we're proving that but there is a lag. So that it's shown a little bit behind the hospital deaths reporting.

*26^th^ April*

The last slide is an international comparison and there are two lines here for the UK, the first is hospital deaths which we can report very quickly, but then also you'll see a line now at around day 24-25 in all settings which includes data that becomes available through the Office for National Statistics. That includes all deaths but because of the way that deaths are reported out of hospitals there is a lag in reporting that, it is updated weekly and we are looking at ways in which we can ensure that that reporting is done quicker but there will inevitably be a lag on that.

*27^th^ April*

Finally a slide which compares different countries and again I would be really clear that we should not over-interpret the absolute numbers the ways these are actually measured in different countries is different but the trend lines I think are reasonably clear from this.

*28^th^ April*

The last slide for today compares UK deaths with those in other nations. So these lines are lined up against the first day when there had been a total of 50 deaths accumulated in each of these nations. And we have two different lines on there describing what's happened in the UK. Towards the right hand side is a dark blue line labelled UK hospitals only and that's the data that we've been collecting and showing for a long time and that is people who died with covid in UK hospitals. Whereas a grey line further over to the left there is people who died with covid in the UK in all settings. And not surprisingly if you count all settings that line is higher than the people who died in UK hospitals.

*29th April – two slides discussed on this day, the first is the equivalent of Figure 1*

Now on to global, so global death comparisons are two slides here the first one is actually just crude numbers, and you can see here a pattern that looks like the United States has a very high number of deaths and other countries are aligning with each other but the point on this slide at the side says a number of things. First of all these are measures may not be comprehensive measures of death in the way that we're - we're very keen to do here and are doing now, comprehensively. So for instance in Spain we're not absolutely clear if the deaths in Spain include care home deaths. And this is understandable because it is very complex and difficult to get these measures particularly on a daily basis, which is why the news today about being able to do this on a daily basis is very good news although of course the news about numbers of death is not good news and again stresses how dangerous this virus is, how sad it is for those individuals and how we have to be ever-vigilant about how we look after each other and how we adhere to the request to stay at home, protect the NHS and particularly to save lives. Now the last slide is also interesting because this now shows the cumulative death number per million population and this shows a very different pattern where we can see two different measures for the UK, the hospital deaths and the comprehensive deaths, not hugely different on this scale. But you can see where we are now tracking much more along our European neighbours and also, we've seen Italy and Spain there and France but the US of course has dropped because we are now measuring rate per million, other countries have increased. The important thing here is that our measures are comprehensive, they're daily and they take in all care settings. So I thought you would wish to see the two contrasts.

*30^th^ April*

And again, this is the comparison, it's difficult to actually look at exactly what this means because people record things differently.

*1^st^ May*

And then very finally we show the usual international comparison against other countries and again this comes with the usual caveat that this sort of comparison is really important it's now UK all settings but the real comparison is in all-cause mortality so in excess deaths in countries that is measured more consistently between countries but there will be some time before that analysis can be done. So although it's important to show this data, I think it's important to remember that it will be a number of months and perhaps longer before we can see the true comparison between countries.

*2^nd^ May – slide is present in presentation material but not discussed in briefing*

*3^rd^ May*

And then in the final slide, as we've shown before, an international comparison now reporting UK deaths in all settings. Remember previously in these charts showing deaths, we were reporting for some time deaths in hospital, this is now all settings. And again, the usual caveats with this, the different countries measure this differently, the measure that I think we all recognise will be the key measure is excess deaths for all mortality because that will take into account the overall effect of the pandemic, but it will be some time before that sort of comparison can be done between countries.

*4^th^ May*

And then the final slide is one we have shown for some time, showing a comparison across countries dating back to the day when 50 cumulative deaths were recorded in each of the countries, and we're now quite away across the slide to 60 odd days in some of the countries. The message is the same as I've said to you before that there are kind of three broad trends on this curve, but I do want to emphasize to you: these are number, they are not rates, they do not adjust for the size of the underlying population and this is a difficulty in interpreting these data. We will in the course of time be able to get all-cause age-adjusted excess mortality for many of the countries on the slide. And that will give us a much clearer understanding of what is going on. And hopefully those data will continue for a long period of time so that we can understand the fullness of the picture as we try to beat this virus.

*5^th^ May*

The final slide for today is the comparison of numbers of deaths for different countries. The UK which is covid deaths in all settings is the grey line in the middle there and what you see is that death is still continuing to climb and is higher than we would wish. I think is all I can say.

*6^th^ May*

And then finally two slides about the international deaths. So the first one is just the crude numbers and this of course depends on the size of the country, so we have over 67 thousand deaths in the US but of course a big country and we can see the UK and the European countries there further down, but if we look at the last slide, which is about the rates per million population then we've taken account of the size of the country, and we can see a different pattern for the UK and the European countries, all are more closely together. I want to say that is really is presented just to show different ways of looking at death, there are many ways of looking at death. It is far too early to say how this will eventually result for how countries have fared in this epidemic. We need to give this some time, most of those who are used to looking at these things would say probably a year, but we thought you would be interested to see.

*7^th^ May*

And then finally just a quick comparison. It's important that we compare data internationally and this is global deaths comparisons, but as we've mentioned frequently there are variations in how different countries measure those deaths and these are numbers rather than rates.

*8^th^ May*

And then last is the international comparison this shows UK all settings now with a lag on it, sorry not with a lag on it, but UK all settings set from a particular start point for all countries. And again, the usual caveats that we give, that comparison between different countries is difficult and it will be only be excess mortality, all-cause mortality over a period of time of the pandemic that will be probably the best way to make this sort of comparison.

*9^th^ May*

And then finally this is the global death comparison that you have seen before. All lines begin on the bottom left-hand corner at a point when 50 cumulative deaths were recorded in each of the countries and it continues to tell the story that I have illustrated to you in previous press conferences that the US is an outlier at the top, that the UK is in company with France, Spain and Italy in the middle band and there are other European countries and South Korea along the lower trajectory. These data are always difficult to interpret. Every time I want to say it that we will not get the most granular picture until we start to get excess mortality data across all of these countries and that will help us make a far more accurate comparison.

**Appendix 2:** Dates, occurrences and counts of statements made in UK Government news briefings which implied that that undertaking cross-country comparisons was challenging

| **Date** | **Difficult + comparison*, compare, comparative** | **Interpret + difficult, difficulty**  **\|over-interpreting** | **Different + measure*, record, system** | **Number* + crude, absolute** | **Number* + rate*** | **Caveat*** | **Excess death*, excess mortality, all-cause mortality, all mortality** | **Size*, population*, adjust, adjusted** | **Some time** | **Comparison + true,**  **accurate,**  **real**  **\|clearer understanding** | **Total** |
| --- | --- | --- | --- | --- | --- | --- | --- | --- | --- | --- | --- |
| 30/03/2020 | 0 | 0 | 0 | 0 | 0 | 0 | 0 | 0 | 0 | 0 | 0 |
| 31/03/2020 | 0 | 0 | 0 | 0 | 0 | 0 | 0 | 0 | 0 | 0 | 0 |
| 01/04/2020 | 0 | 0 | 0 | 0 | 0 | 0 | 0 | 0 | 0 | 0 | 0 |
| 02/04/2020 | 0 | 0 | 0 | 0 | 0 | 0 | 0 | 0 | 0 | 0 | 0 |
| 03/04/2020 | 0 | 0 | 0 | 0 | 0 | 0 | 0 | 0 | 0 | 0 | 0 |
| 04/04/2020 | 0 | 0 | 0 | 0 | 0 | 0 | 0 | 0 | 0 | 0 | 0 |
| 05/04/2020 | 0 | 1 | 0 | 0 | 0 | 0 | 0 | 0 | 0 | 0 | 1 |
| 06/04/2020 | 0 | 0 | 0 | 0 | 0 | 0 | 0 | 0 | 0 | 0 | 0 |
| 07/04/2020 | 0 | 0 | 0 | 0 | 0 | 0 | 0 | 0 | 0 | 0 | 0 |
| 08/04/2020 | 0 | 0 | 0 | 0 | 0 | 0 | 0 | 0 | 0 | 0 | 0 |
| 09/04/2020 | 0 | 0 | 0 | 0 | 0 | 0 | 0 | 0 | 0 | 0 | 0 |
| 10/04/2020 | 0 | 0 | 0 | 0 | 0 | 0 | 0 | 0 | 0 | 0 | 0 |
| 11/04/2020 | 0 | 0 | 0 | 0 | 0 | 0 | 0 | 0 | 0 | 0 | 0 |
| 12/04/2020 | 0 | 0 | 0 | 0 | 0 | 0 | 0 | 0 | 0 | 0 | 0 |
| 13/04/2020 | 0 | 0 | 0 | 0 | 0 | 0 | 0 | 0 | 1 | 0 | 1 |
| 14/04/2020 | 0 | 0 | 0 | 0 | 0 | 0 | 0 | 0 | 0 | 0 | 0 |
| 15/04/2020 | 0 | 0 | 0 | 0 | 0 | 0 | 0 | 0 | 0 | 0 | 0 |
| 16/04/2020 | 0 | 0 | 0 | 0 | 0 | 0 | 0 | 0 | 0 | 0 | 0 |
| 17/04/2020 | 0 | 0 | 0 | 0 | 0 | 0 | 0 | 0 | 0 | 0 | 0 |
| 18/04/2020 | 0 | 0 | 0 | 0 | 0 | 0 | 0 | 0 | 0 | 0 | 0 |
| 19/04/2020 | 0 | 0 | 1 | 0 | 0 | 0 | 0 | 0 | 0 | 0 | 1 |
| 20/04/2020 | 0 | 0 | 0 | 0 | 0 | 0 | 0 | 0 | 0 | 0 | 0 |
| 21/04/2020 | 0 | 0 | 0 | 0 | 0 | 0 | 2 | 0 | 0 | 0 | 2 |
| 22/04/2020 | 1 | 0 | 0 | 0 | 0 | 0 | 0 | 0 | 0 | 0 | 1 |
| 23/04/2020 | 0 | 0 | 0 | 0 | 0 | 0 | 0 | 0 | 0 | 0 | 0 |
| 24/04/2020 | 1 | 0 | 0 | 0 | 0 | 0 | 0 | 0 | 0 | 0 | 1 |
| 25/04/2020 | 0 | 0 | 0 | 0 | 0 | 0 | 0 | 0 | 0 | 0 | 0 |
| 26/04/2020 | 0 | 0 | 0 | 0 | 0 | 0 | 0 | 0 | 0 | 0 | 0 |
| 27/04/2020 | 0 | 1 | 1 | 1 | 0 | 0 | 0 | 0 | 0 | 0 | 3 |
| 28/04/2020 | 0 | 0 | 0 | 0 | 0 | 0 | 0 | 0 | 0 | 0 | 0 |
| 29/04/2020 | 0 | 0 | 1 | 1 | 0 | 0 | 0 | 1 | 0 | 0 | 3 |
| 30/04/2020 | 0 | 1 | 1 | 0 | 0 | 0 | 0 | 0 | 0 | 0 | 2 |
| 01/05/2020 | 0 | 0 | 0 | 0 | 0 | 1 | 1 | 0 | 1 | 2 | 5 |
| 02/05/2020 | 0 | 0 | 0 | 0 | 0 | 0 | 0 | 0 | 0 | 0 | 0 |
| 03/05/2020 | 0 | 0 | 1 | 0 | 0 | 1 | 2 | 0 | 1 | 0 | 5 |
| 04/05/2020 | 0 | 1 | 0 | 0 | 1 | 0 | 1 | 4 | 1 | 1 | 9 |
| 05/05/2020 | 0 | 0 | 0 | 0 | 0 | 0 | 0 | 0 | 0 | 0 | 0 |
| 06/05/2020 | 0 | 0 | 0 | 1 | 0 | 0 | 0 | 3 | 1 | 0 | 5 |
| 07/05/2020 | 0 | 0 | 1 | 0 | 1 | 0 | 0 | 0 | 0 | 0 | 2 |
| 08/05/2020 | 1 | 0 | 0 | 0 | 0 | 1 | 2 | 0 | 0 | 0 | 4 |
| 09/05/2020 | 0 | 1 | 0 | 0 | 0 | 0 | 1 | 0 | 0 | 1 | 3 |

**Notes:** We used the software “AntConc” to search for phrases (Anthony, 2019). Here, we use the symbols in column headings to denote how the search was conducted. “*” indicates any character could follow; “,” acts as an OR; “+” operates as an AND; | indicates that term alone would be sufficient. The phrase “some time” is included as it is used in the context of needing longer before results would be known.

**Appendix 3:** Linear regression of total Covid-19 deaths per million (as of 9^th^ June)

|  | Estimate | Std. Error | t value | Pr(>\|t\|) |
| --- | --- | --- | --- | --- |
| Constant | -48.779 | 576.741 | -0.085 | 0.933 |
| Population density (people per km^2)^ | 0.060 | 0.292 | 0.206 | 0.838 |
| GDP per capita (PPP$) | 0.002 | 0.003 | 0.808 | 0.426 |
| Gini coefficient | 4.422 | 7.224 | 0.612 | 0.545 |
| Urban population (%) | 2.379 | 3.420 | 0.696 | 0.492 |
| Warning days | -7.687 | 5.816 | -1.322 | 0.196 |
| Aged 70+ (%) | 9.930 | 13.838 | 0.718 | 0.479 |
| Residual standard error: 202.7 on 30 degrees of freedom  Multiple r-squared: 0.2315, Adjusted r-squared: 0.0778  F-statistic: 1.506 on 6 and 30 DF, p-value: 0.21 | | | | |

**Notes:** Regressors are country specific variables thought to influence death toll. Note that while some variation is explained (~25%) the vast majority is unexplained by these factors. Data from most variables are from Our World in Data (Beltekian et al., 2020). This is supplemented by data from the World Bank for the Gini Coefficient (downloaded from: <https://data.worldbank.org/indicator/SI.POV.GINI?view=map>) and the percentage of the population which is urban (downloaded from: <https://data.worldbank.org/indicator/SP.URB.TOTL.IN.ZS>). Data is missing for the New Zealand Gini coefficient hence we use the figure published by their government (downloaded from: <https://www.stats.govt.nz/information-releases/household-income-and-housing-cost-statistics-year-ended-june-2019#download-data>).

**Appendix 4:** Lockdown dates by country.

| **Country** | **Lockdown imposed** | **Lockdown eased** | **Length of lockdown (days)** |
| --- | --- | --- | --- |
| **Belgium** | 18/03/2020 | 04/05/2020 | 47 |
| **China** (Hubei) | 23/01/2020 | 25/03/2020 | 62 |
| **Denmark** | 17/03/2020 | 14/04/2020 | 28 |
| **Germany** | 21/03/2020 | 27/04/2020 | 37 |
| **Italy** | 08/03/2020 | 04/05/2020 | 57 |
| **Korea** | 21/03/2020 | 19/04/2020 | 29 |
| **New Zealand** | 23/03/2020 | 27/04/2020 | 35 |
| **United Kingdom** | 23/03/2020 | 11/05/2020 | 49 |
| **United States** | 22/03/2020 | [24/06/2020] | [94] |

**Notes:** Dates are typically the first time a country imposed strict (at least level 2) restrictions on internal movement, taken from the Oxford Government Response Tracker (Hale et al., 2020). These are very similar to the dates used by Flaxman et al (2020). The lockdown date used for Italy is when multiple provinces in the north of the country locked down; some small towns had been quarantined before, the rest of the country was locked down the following day (9^th^ March). Denmark never imposed quite such strict requirement; the lockdown date comes from Flaxman et al. The dates for lockdown easement come from various newspaper sources. For the United States, the date used for lockdown emerges from the data; different states locked down at different times, but this is the best fit to the pattern of infection. Likewise, the US has been easing lockdown regionally. The SEIR model does not assume lockdown is lifted, but for the financial cost we must, and so use the end of projection as the assumed date for lifting lockdown.

**Appendix 5:** R values – posterior estimates from the Bayesian modelling for each country, and prior assumptions

| **Country** | **R0**  Mean  [95% CIs] | **R1**  Mean  [95% CIs] | **R2**  Mean  [95% CIs] |
| --- | --- | --- | --- |
| **Prior** | N(3,1^2^) | N(1,0.5^2^) | N(1,0.2^2^) |
| **Belgium** | 3.75  [1.93, 5.50] | 0.75  [0.64, 0.86] | 0.87  [0.60, 1.15] |
| **China** | 3.08  [1.73; 4.58] | 0.54  [0.41; 0.68] | 0.98  [0.77; 1.22] |
| **China** (Hubei) | 3.13  [1.38, 4.73] | 0.58  [0.47, 0.73] | 1.02  [0.75, 1.26] |
| **Denmark** | 2.77  [1.12, 4.78] | 0.64  [0.34, 0.99] | 0.87  [0.67, 1.11] |
| **Germany** | 3.30  [2.24, 4.62] | 0.69  [0.55, 0.85] | 0.86  [0.59, 1.10] |
| **Italy** | 3.41  [2.04, 4.85] | 0.77  [0.66, 0.91] | 1.00  [0.71, 1.31] |
| **Korea** | 2.42  [0.72, 3.87] | 0.72  [0.53, 0.93] | 0.98  [0.75, 1.19] |
| **New Zealand** | 3.09  [1.61, 4.83] | 0.91  [0.63, 1.33] | 1.01  [0.64, 1.36] |
| **United Kingdom** | 3.48  [2.28, 5.17] | 0.83  [0.73, 0.96] | 0.87  [0.57, 1.15] |
| **United States** | 3.49  [2.49, 4.99] | 0.92  [0.84, 0.99] | N/A |

**Notes:** R values are estimated using Bayesian updating as described in text. R0 corresponds to the infection rate pre-lockdown, R1 during lockdown, and R2 post-lockdown. All R values relate to the fundamental model parameter that describes the infection number in a naive population. “Effective R” values late in the epidemics will be lower by around 0-10% to account for the proportion of the population that has been infected.

**Appendix 6:** Predicted and actual daily deaths for each country.


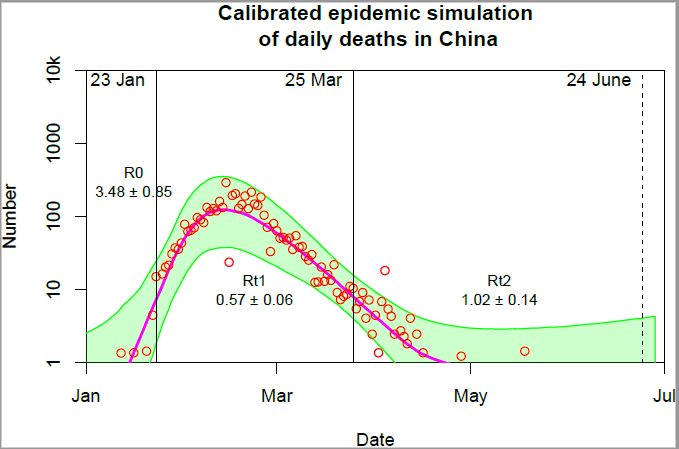

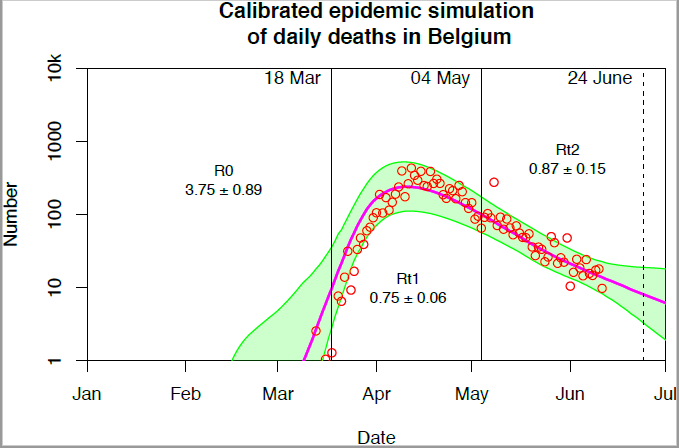
The key dates with respect to lockdown are indicated by the vertical lines and accompanying text. The posterior effective reproductive rate, R, is also show, along with the 95% CIs.

**Notes:** This graph is for just Hubei province, China


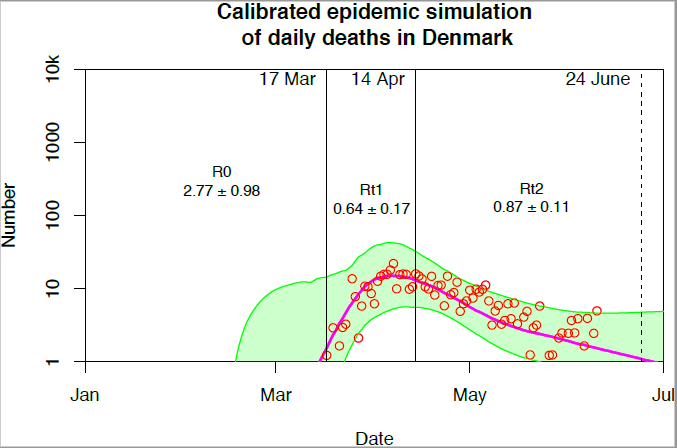


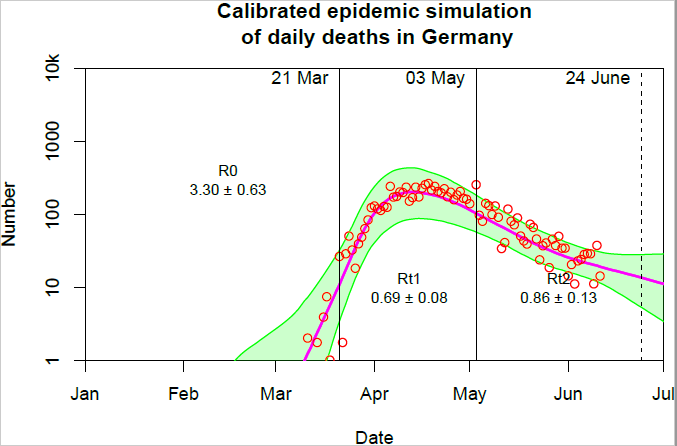


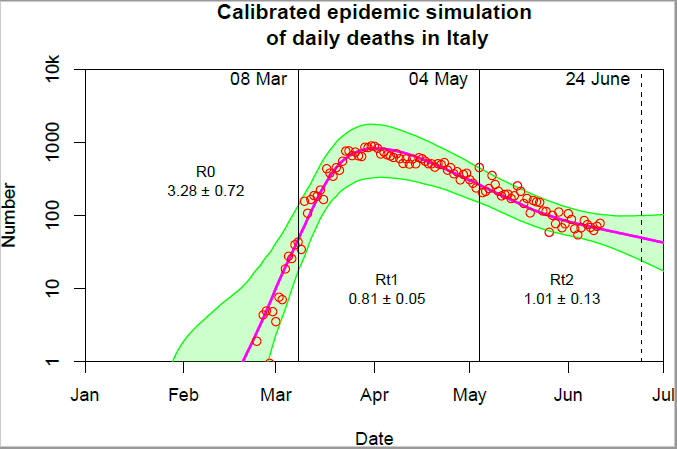


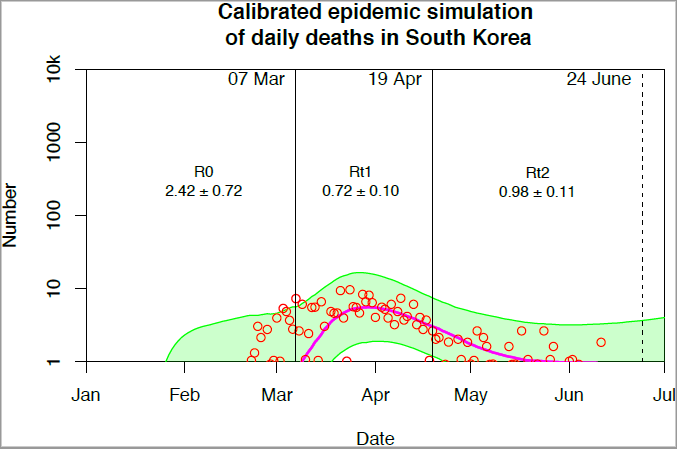


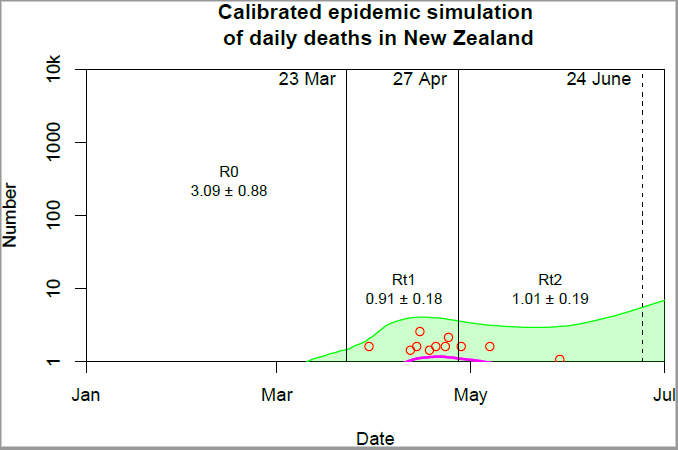


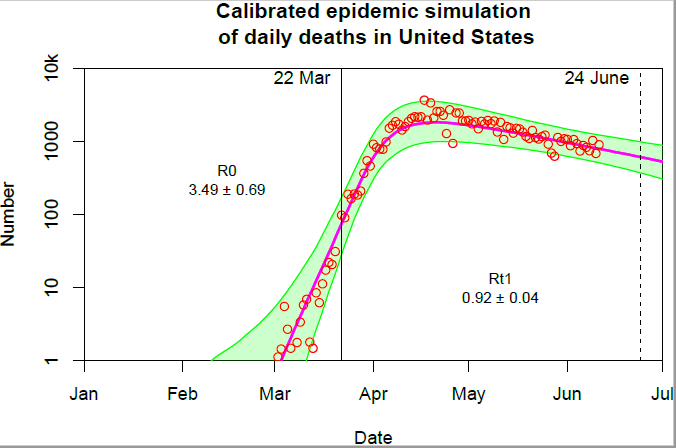


**Notes:** The USA has yet to “unlock” hence the lack of a right hand solid line.

**Appendix 7:** Difference in lives saved (lost) by earlier (later) lockdown compared with baseline.

| **Country** | **7 days earlier**  Mean  [95% CIs] | **7 days later**  Mean  [95% CIs] | **12 days earlier**  Mean  [95% CIs] | **12 days later**  Mean  [95% CIs] |
| --- | --- | --- | --- | --- |
| **Belgium** | 7,500  [3,600; 13,800] | 23,200  [7,200; 43300] | 8,900  [4,400 16,200] | 44,900  [13,900; 77,000] |
| **China** (Hubei) | 3,500  [900; 8,300] | 13,700  [2,300; 34,000] | 4,100  [1,100 9,600] | 30,900  [5,500; 62,000] |
| **Denmark** | 400  [100; 1,000] | 1,900  [200; 6,000] | 600  [200; 1,100] | 5,700  [300; 18,500] |
| **Germany** | 6,500  [3,200; 11,700] | 30,600  [10,200; 73,400] | 7,600  [4,000; 13,400] | 94,100  [28,300; 230,600] |
| **Italy** | 30,800  [11,700; 55,900] | 87,900  [28,700; 174,300] | 37,300  [15,700; 68,200] | 182,000  [60,500; 336,600] |
| **Korea** | 183  [27; 407] | 670  [30; 2121] | 233  [45; 512] | 2,277  [52; 9,970] |
| **New Zealand** | 60  [19; 108] | 296  [38; 826] | 73  [25; 126] | 1,105  [78; 3,652] |
| **United Kingdom** | 33,900  [16,000; 64,400] | 98,300  [38,300; 182,000] | 40,900  [20,500; 77,200] | 203,800  [83,900; 379,300] |
| **United States** | 84,900  [54,200; 137,500] | 295,900  [131,500; 546,700] | 101,500  [69,100; 160,800] | 711,100  [297,900; 1,319,000] |

**Notes:** Estimates are rounded the nearest hundred, other than for Korea and New Zealand where, owing to their early intervention, enacting slightly earlier or later lockdowns would make relatively little difference to the number of deaths for each country.


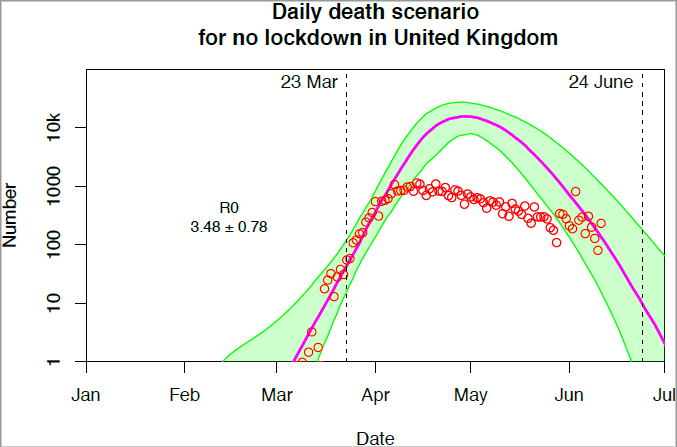
**Appendix 8:** Modelled daily deaths for the UK if lockdown had not occurred

**Notes:** The solid line (shaded area) shows the predicted (95% CI) trajectory of deaths in the UK had lockdown not been enacted instead of being imposed on 23^rd^ March (left hand dotted line). Red dots indicate actual daily deaths in the UK. The reproductive rate is that which is calibrated on the data for the pre-lockdown trend.

| **Country** | **GDP ($bn, PPP)** | **ΔIMF GDP forecast (%)** | **Actual lockdown (days)** | **GDP consequence 3 day change ($bn, PPP)** | **GDP consequence 7 day change ($bn, PPP)** | **GDP consequence 12 day change ($bn, PPP)** |
| --- | --- | --- | --- | --- | --- | --- |
| **Belgium** | 540 | -8.2 | 47 | 0.4 | 1.0 | 1.7 |
| **China** (Hubei) | 1,290 | -4.6 | 62 | 0.4 | 1.0 | 1.7 |
| **Denmark** | 300 | -8.4 | 28 | 0.4 | 0.9 | 1.6 |
| **Germany** | 4,160 | -8.2 | 37 | 4.1 | 9.7 | 16.6 |
| **Italy** | 2,250 | -9.7 | 57 | 1.7 | 4.0 | 6.9 |
| **Korea** | 2,310 | -3.4 | 29 | 1.2 | 2.8 | 4.9 |
| **New Zealand** | 200 | -9.9 | 35 | 0.2 | 0.6 | 1.0 |
| **United Kingdom** | 2,980 | -8.0 | 49 | 2.2 | 5.1 | 8.7 |
| **United States** | 20,290 | -8.0 | 94 | 7.8 | 18.1 | 31.1 |

**Appendix 9:** Financial ramifications of extended lockdowns

**Notes:** Data are form the IMF in columns 2 and 3. Lockdown length is from Appendix 4. Columns 5-7 are the results of our calculations as per Equation 2. The financial consequence of a shortened lockdown (delayed implementation) are shown. Earlier lockdown represents a cost to the economy of the same magnitude.

**Appendix 10:** Implied price of life in different countries using different perturbations to lockdown imposition.

| **Country** | **Accepted price of life (7 days)**  Mean  [95% CIs] | **Rejected price of life (7 days)**  Mean  [95% CIs] | **Accepted price of life (12 days)**  Mean  [95% CIs] | **Rejected price of life (12 days)**  Mean  [95% CIs] |
| --- | --- | --- | --- | --- |
| **Belgium** | 43,000  [23,000; 137,000] | 131,000  [71,000; 275,000] | 38,000  [22,000; 122,000] | 190,000  [104,000; 386,000] |
| **China** (Hubei) | 74,000  [30,000; 440,000] | 293,000  [122,000; 1,175,000] | 56,000  [28,000; 316,000] | 429,000  [181,000; 1,607,000] |
| **Denmark** | 498,000  [158,000; 6,153,000] | 2,112,000  [964,000; 7,598,000] | 285,000  [88,000; 5,760,000] | 2,946,000  [1,413,000; 8,548,000] |
| **Germany** | 317,000  [132,000; 950,000] | 1,497,000  [826,000; 3,000,000] | 176,000  [72,000; 586,000] | 2,176,000  [1,238,000; 4172,000] |
| **Italy** | 45,000  [23,000; 139,000] | 130,000  [72,000; 342,000] | 38,000  [20,000; 113,000] | 184,000  [101,000; 437,000] |
| **Korea** | 4,235,000  [1,338,000; 94,585,000] | 15,472,000  [6,967,000; 104,768,000] | 2,136,000  [488,000; 93,545,000] | 20,908,000  [9,499,000; 108,029,000] |
| **New Zealand** | 1,958,000  [702,000; 15,251,000] | 9,606,000  [5,375,000; 30,729,000] | 899,000  [272,000; 12,737,000] | 13,649,000  [7,855,000; 39,529,000] |
| **United Kingdom** | 52,000  [28,000; 132,000] | 149,000  [79,000; 317,000] | 43,000  [23,000; 104,000] | 213,000  [113,000; 424,000] |
| **United States** | 61,000  [33,000; 138,000] | 213,000  [132,000; 334,000] | 44,000  [24,000; 104,000] | 306,000  [193,000; 450,000] |

**Notes:** These results use changes in lives lost and financial estimates associated with a 7 or 12 day perturbation in the lockdown date. Accepted price of life calculated as the trade-off between GDP and life imagining lockdown had been imposed 7 or 12 days later; rejected price of life as if lockdown had been imposed 7 or 12 days earlier. Prices to the nearest thousand
